# Contraceptive use and unintended pregnancy among young women and men in Accra, Ghana

**DOI:** 10.1371/journal.pone.0201663

**Published:** 2018-08-17

**Authors:** Kate Grindlay, Phyllis Dako-Gyeke, Thoai D. Ngo, Gillian Eva, Leonard Gobah, Sarah T. Reiger, Sruthi Chandrasekaran, Kelly Blanchard

**Affiliations:** 1 Ibis Reproductive Health, Cambridge, Massachusetts, United States of America; 2 Department of Social and Behavioral Sciences, School of Public Health, College of Health Sciences, University of Ghana, Accra, Ghana; 3 Poverty, Gender and Youth Program, Population Council, New York, New York, United States of America; 4 Marie Stopes International US, Washington, DC, United States of America; 5 Marie Stopes Ghana, Accra, Ghana; University of Westminster, UNITED KINGDOM

## Abstract

The objective of this study was to determine factors associated with modern contraceptive use and unintended pregnancy among young women and men in Accra, Ghana. From September-December 2013, we conducted a cross-sectional survey with 250 women and 100 men aged 18–24. We explored determinants of modern contraceptive use among males and females and unintended pregnancy among females. Descriptive statistics, chi-square tests, Fisher’s exact tests, and multivariable logistic regression were used. Participants had an average of three lifetime sexual partners, and 91% had one current partner. Overall, 44% reported current modern contraceptive use. In multivariate modeling, modern contraceptive use was associated with higher education compared to primary (AORs 2.1–4.3); ever talking with someone about contraception (AOR 4.7); feeling unsupported by a healthcare provider for contraception (AOR 2.2); and not feeling at risk of unintended pregnancy (AOR 2.7). While ≥70% of participants recognized most contraceptive methods, awareness of some methods was lacking. Nearly all respondents (91%) felt at least one modern method was unsafe. Nearly half of all females (45%) reported their last pregnancy was unintended, and 63% of females and 58% of males felt at risk for future unintended pregnancy. Women were more likely to experience unintended pregnancy if they had ever given birth (AOR 6.7), their sexual debut was 8–14 years versus 20–24 years (AOR 3.4), or they had 3–4 lifetime sexual partners versus 1–2 (AOR 2.4). Targeted interventions are needed to improve understanding of the safety of modern contraceptive methods, increase awareness of long-acting methods, and consequently increase modern contraceptive access and use.

## Introduction

Over half (53%) of the 26.4 million people in Ghana live in urban areas, and by 2050 the proportion is projected to increase to nearly three-quarters (70%) [1]. Young adults aged 15–24 comprise 20% of the country [2]. Adolescence to young adulthood is an important period for physical and mental development, and behaviors during this time can have long term implications. In order to have safe, healthy sexual and reproductive lives, young people need access to comprehensive reproductive health services; however, many face challenges accessing and utilizing the resources they require.

According to the 2014 Ghana Demographic and Health Survey, 39% of Ghanaian women had given birth by age 20. Fifty-one percent of married females aged 15–19 and 34% aged 20–24 had an unmet need for family planning. Six percent of married and sexually active unmarried 15–19 year olds and 21% of married and sexually active unmarried 20–24 year olds were using any modern contraceptive method. More than half (58%) of all births in the prior five years to Ghanaian women aged 15–19 and one-third of births to those aged 20–24 were unintended [3].

Globally, 40% of pregnancies are unwanted, of which 50% end in induced abortion [4]. In developing regions, 214 million women have an unmet contraceptive need, with the highest proportion of women in Sub-Saharan Africa (21%) [5]. While deeply embedded in other sociopolitical barriers, contraceptive non-use and use of traditional methods (withdrawal and rhythm) especially put women at high risk of unintended pregnancy. Unintended pregnancies are associated with negative health, social, and economic outcomes for both the woman and child, and it is estimated that between one-fourth and two-fifths of maternal deaths could be averted if unplanned pregnancies were prevented [4, 6]. Serving all women who currently have an unmet need for modern contraception in developing countries could prevent 67 million unintended pregnancies, 23 million unplanned births, and 36 million abortions, and 76,000 maternal deaths each year [5].

The benefits of preventing unintended pregnancies, particularly among young women, span social, health, and economic domains [7, 8]. Recognizing the potential for significant negative impacts of unintended pregnancy, the Reducing Maternal Mortality and Morbidity program was launched in Ghana in 2006 to improve access to family planning and comprehensive abortion care services [9].

However, studies have found that perceptions about side effects and attitudinal factors pose a challenge to increased family planning use in Ghana [10]. Focus group discussions from a hospital in Legon, Ghana found women’s concern with menstrual regularity results in dissatisfaction with methods that prevent menstruation [11]. Another study found that Ghanaian women perceive family planning as ineffective or unsafe [12], and Ghana Demographic and Health Survey data from 1988 to 2008 show that attitudinal resistance has been an increasing component of unmet need in Ghana [13]. A follow-up study to the 2014 Ghana Demographic and Health Survey showed that women were averse to using modern methods due to risk of side effects, personal or partner opposition to family planning, and religious views [10]. Male attitudes toward contraception are mixed: in the 2014 Ghana Demographic and Health Survey, 73% of men aged 15–59 rejected the idea that contraception is a woman’s business and men should not have to be involved, but 46% supported the statement that women who use contraception may become promiscuous [3]. Husbands’ attitudes toward family planning can remain a barrier to its use; married Ghanaian women’s sexual empowerment is a statistically significant predictor of contraceptive use, even after controlling for other factors [14].

Despite efforts to expand sexual and reproductive health services in Ghana, little is known about young people’s participation [15] or factors contributing to contraceptive use and unintended pregnancy, especially among young men. Few studies in the past decade have explored these topics, and most were qualitative or focused on specific contraceptive methods [16–20]. There is particularly little known about the determinants of young people’s sexual and reproductive health in Ghana. In order to effectively address the reproductive health needs of urban youth, a growing population in Ghana, this study aimed to assess factors associated with modern contraceptive use and unintended pregnancy among young women and men in Accra.

## Materials and methods

### Study design and sample

From September to December 2013, we conducted a cross-sectional survey with young women and men aged 18–24 in Accra, Ghana (dx.doi.org/10.17504/protocols.io.rkyd4xw [PROTOCOL DOI]). We employed a stratified random sampling technique among male/female strata in two low- and middle-income communities in greater Accra [21]: Kokomlemle, a busy trading area, and Tema New Town, a fishing community. These two sites were chosen because they would be representative of Accra, more broadly.

The sample size for females was determined based on a primary outcome, current contraception use, modeled using logistic regression. We anticipated 21% of female participants would use a method of contraception [3]. Using the rule of thumb that ten observations are needed per degree of freedom [22], a multivariable model with five degrees of freedom could be reliably fitted with a female sample size of 250. The data for the male component of the survey were exploratory and sample size was based on feasibility.

In-person survey interviews were conducted in private locations near recruitment sites, including market places, social clubs, and sports venues. Teams of paired, trained field workers of the same sex and approximately same age as participants administered surveys. Field workers were students of public health who had prior data collection experience. For this study, they were specifically trained on the project protocol, research ethics, sampling strategy, and administration of data collection tools. Following the training, field workers were required to pilot data collection instruments and provided feedback.

Eligibility to participate in the study included being male or female, aged 18–24, speaking English, Twi, or Ga, and having had sexual intercourse within six months prior to the survey. Field workers approached young people in the recruitment sites, introduced the study, and requested them to participate if interested. Eligible and interested respondents were given a copy of the informed consent form, which outlined the background of the study, possible risks and benefits, confidentiality measures, and contacts for additional information. Participants were also informed that participation was completely voluntary and that they were free to not answer any questions and/or stop the survey at any point. Field workers obtained written informed consent from participants. Surveys took approximately 45–60 minutes, and participants received ten Ghanaian Cedi ($3.00 USD) as an appreciation of their time.

Study instruments were piloted prior to data collection. We received IRB approval from two Institutional Review Boards—the Institutional Review Board of the Nouguchi Memorial Institute for Medical Research at the University of Ghana, Legon, and the Marie Stopes International Ethical Review Committee. Study instruments were in English, but questions were translated in Twi or Ga by field workers when needed.

### Measures

Sociodemographic measures included age, education, religion, and relationship status. Reproductive health measures included age of sexual debut, current and lifetime number of sexual partners, pregnancy history (including unintended pregnancy), and history of preventive health screenings. Contraceptive measures included ability to spontaneously name or recognize upon surveyor prompt contraceptive methods, perceptions of contraceptive safety and pregnancy risk, opinions about family size, current contraceptive use, sources, satisfaction, support, and reasons for non-use. We also asked about participants’ experiences with abortion, and these data are reported separately.

Modern contraceptive use among males and females (1 = current modern method use reported, 0 = no method or traditional method use reported) was assessed by the question: “Are you [and your partner] currently doing something or using any method to delay or avoid getting pregnant?” Participants who reported ‘yes’ were then asked to list all current methods. We categorized current contraceptive method use according to the most effective method reported [23] and responses were coded as ‘none,’ ‘traditional method’ (defined as rhythm or withdrawal methods), or ‘modern method’ (defined as female and male sterilization, intrauterine device (IUD), injectable, implant, pill, male or female condom, lactational amenorrhea method, diaphragm, foam/jelly, or emergency contraception). Unintended pregnancy (1 = last pregnancy unintended, 0 = never pregnant or last pregnancy intended) among females was assessed by the question: “At the time you [last] became pregnant, did you want to become pregnant then, did you want to wait until later, or did you not want to have (more) children at all?” Those who reported ‘later’ or ‘not at all’ were coded as having an unintended pregnancy.

### Analysis

All analyses were conducted using Stata 12.1 (Stata, StataCorp, College Station, TX) and statistical tests assumed significance at p-value <0.05. Descriptive statistics, chi-square tests, and Fisher’s exact tests were used. Logistic regression was performed to analyze determinants of modern contraceptive use among males and females and unintended pregnancy among females. All variables were used as binary or categorical predictors, with one category selected as the reference group based on large sample size and/or meaningful comparison. Missing data were excluded from these analyses. Socio-demographic and reproductive characteristic variables from Table 1 and contraceptive attitude support variables from Table 2 were included in initial regression models. Sequentially, extraneous variables with a p-value >0.20 were removed from the model.

**Table 1:** Sociodemographic and sexual and reproductive health characteristics, by sex.

	Total sample		Female		Male		P-value
Characteristics	N	%	n	%	n	%	
Total sample size	N = 350	100%	n = 250	71.4%	n = 100	28.6%	
**Sociodemographic characteristics**							
Age	(Mean 21.0, IQR 4.0)		(Mean 21.1, IQR 4.0)		(Mean 20.7, IQR 3.0)		0.45
*18–20 years*	150	42.9%	104	41.6%	46	46.0%	
*21–24 years*	200	57.1%	146	58.4%	54	54.0%	
Highest level of education							**≤0.001**
*JHS*	178	50.9%	138	55.2%	40	40.0%	
*SHS*	95	27.1%	59	23.6%	36	36.0%	
*Tertiary*	17	4.9%	13	5.2%	4	4.0%	
*Vocational/technical*	32	9.1%	13	5.2%	19	19.0%	
*None*	28	8.0%	27	10.8%	1	1.0%	
Religion^a^							0.08
*Christian*	285	81.7%	202	81.1%	83	83.0%	
*Muslim*	60	17.2%	46	18.5%	14	14.0%	
*Other (no religion*, *traditional*, *spiritualist)*	4	1.2%	1	0.4%	3	3.0%	
Relationship status							**≤0.001**
*Married or cohabitating with a stead partner*	79	22.6%	71	28.4%	8	8.0%	
*Steady partner*, *not cohabitating*	240	68.6%	167	66.8%	73	73.0%	
*Single*, *separated*, *divorced*, *or widowed*	31	8.9%	12	4.8%	19	19.0%	
**Sexual and reproductive health history**							
Age of sexual debut^a^	(Mean 16.9, IQR 3.5)		(Mean 17.2, IQR 3.0))		(Mean 16.3, IQR 3.0)		0.06
*8–14 years*	53	15.2%	35	14.1%	18	18.2%	
*15–19 years*	247	71.0%	173	69.5%	74	74.8%	
*20–24 years*	48	13.8%	41	16.5%	7	7.1%	
Lifetime number of sexual partners^a^	(Mean 3.02, IQR 2.0)		(Mean 2.22, IQR 2.0)		(Mean 5.01, IQR 4.0)		**≤0.001**
*1*	96	27.5%	83	33.3%	13	13.0%	
*2*	110	31.5%	94	37.8	16	16.0%	
*3–4*	91	26.1%	56	22.5%	35	35.0%	
*5+*	52	14.9%	16	6.4%	36	36.0%	
Current number of sexual partners^a^	(Mean 1.0, IQR 0.0)		(Mean 1.0, IQR 0.0)		(Mean 1.1, IQR 0.0)		**≤0.001**
*0*	16	4.6%	5	2.0%	11	11.0%	
*1*	317	91.1%	242	97.6%	75	75.0%	
*2+*	15	4.3%	1	0.4%	14	14.0%	
Ever pregnant							**≤0.001**
*Yes*	173	49.4%	154	61.6%	19	19.0%	
*No*	177	50.6%	96	38.4%	81	81.0%	
Ever given birth or fathered child							**≤0.001**
*Yes*	114	32.6%	104	41.6%	10	10.0%	
*No*	236	67.4%	146	58.4%	90	90.0%	
Ever had abortion or partner ever had abortion							**0.002**
*Yes*	88	25.1%	74	29.6%	14	14.0%	
*No or don’t know*	262	74.9%	176	70.4%	86	86.0%	
Last pregnancy unplanned^b^							n/a
*Yes*			113	45.2%			
*No or never pregnant*			137	54.8%			
Ever tested for a sexually transmitted infection^a^							**≤0.001**
*Yes*	131	37.5%	110	44.2%	21	21.0%	
*No*	218	62.5%	139	55.8%	79	79.0%	
Ever had cervical cancer screening^a^^,^^b^							n/a
*Yes*			6	2.4%			
*No or don’t know*			243	97.6%			

^a^Columns may not sum to total sample due to missing data.

^b^Men not asked this question.

Bolded values indicate p<0.05; n/a—not applicable; IQR- interquartile range

**Table 2:** Awareness of and attitudes toward contraceptive methods, by sex.

	Total sample (N = 350)		Female (n = 250)		Male (n = 100)		P-value
Method awareness	N	%	n	%	n	%	
Female sterilization							**0.002**
*Yes*	245	70.0%	163	65.2%	82	82.0%	
*No*	105	30.0%	87	34.8%	18	18.0%	
Male sterilization							**0.001**
*Yes*	190	54.3%	122	48.8%	68	68.0%	
*No*	160	45.7%	128	51.2%	32	32.0%	
Pill							0.08
*Yes*	333	95.1%	241	96.4%	92	92.0%	
*No*	17	4.9%	9	3.6%	8	8.0%	
Intrauterine device^a^							**0.006**
*Yes*	203	59.7%	158	64.2%	45	47.9%	
*No*	137	40.3%	88	35.8%	49	52.1%	
Implant^a^							**≤0.001**
*Yes*	121	35.8%	72	28.8%	49	55.7%	
*No*	217	64.2%	178	71.2%	39	44.3%	
Injectables^a^							**0.002**
*Yes*	326	93.4%	240	96.0%	86	86.9%	
*No*	23	6.6%	10	4.0%	13	13.1%	
Male condom							n/a
*Yes*	350	100.0%	250	100.0%	100	100.0%	
*No*	0	0.0%	0	0.0%	0	0.0%	
Female condom^a^							**0.001**
*Yes*	316	90.8%	217	87.5%	99	99.0%	
*No*	32	9.2%	31	12.5%	1	1.0%	
Diaphragm^a^							0.26
*Yes*	85	25.1%	65	26.8%	20	20.8%	
*No*	254	74.9%	178	73.3%	76	79.2%	
Foam/jelly^a^							**≤0.001**
*Yes*	158	45.4%	98	39.2%	60	61.2%	
*No*	190	54.6%	152	60.8%	38	38.8%	
Lactational amenorrhea method^a^							**0.01**
*Yes*	71	21.9%	44	18.4%	27	31.4%	
*No*	254	78.2%	195	81.6%	59	68.6%	
Rhythm method^a^							**0.01**
*Yes*	328	94.0%	229	92.0%	99	99.0%	
*No*	21	6.0%	20	8.0%	1	1.0%	
Withdrawal							**0.07**
*Yes*	342	97.7%	242	96.8%	100	100.0%	
*No*	8	2.3%	8	3.2%	0	0.0%	
Emergency contraception^a^							0.70
*Yes*	306	87.9%	220	88.4%	86	86.9%	
*No*	42	12.1%	29	11.6%	13	13.1%	
**Contraceptive attitudes**							
Women who use contraception may become promiscuous^a^	136	39.7%	83	34.0%	53	53.5%	**0.001**
In general thinks contraception is risky for women’s health^a^	92	26.7%	38	15.6%	54	54.0%	**≤0.001**
Thinks any of the modern methods are unsafe for women’s health^a^	316	90.5%	217	87.2%	99	99.0%	**0.001**
Children in smaller families are more likely to succeed^a^	295	85.8%	199	81.6%	96	96.0%	**0.001**
Feels somewhat or very likely to get pregnant accidentally^a^	208	61.2%	150	62.5%	58	58.0%	0.44
**Support for contraceptive use**							
Ever talked with anyone about contraceptive use^a^	201	57.9%	134	54.3%	67	67.0%	**0.03**
Partner support^a^^,^^b^			147	59.3%			n/a
Friend support^a^	203	58.7%	146	59.3%	57	57.0%	0.69
Family support^a^	94	27.2%	75	30.5%	19	19.0%	**0.03**
Healthcare provider support^a^	176	50.9%	134	54.5%	42	42.0%	**0.04**

^a^Columns may not sum to total sample due to missing data.

^b^Men not asked this question.

Bolded values indicate p<0.05; n/a—not applicable

## Results

### Demographic and reproductive health characteristics

Overall, 100 males and 250 females participated in the survey. Sociodemographic and sexual and reproductive health characteristics, by sex, are presented in Table 1. Study participants were on average 21 years old (interquartile range: 4.0), unmarried with a steady non-cohabitating partner (67% female, 73% male), and Christian (81% female, 83% male). Males had significantly higher levels of education compared to females: 60% of males had senior high school education or higher compared to 34% of females (p≤0.001).

Average age of sexual debut was 17 for females and 16 for males, and on average male and female participants had been sexually active for four years. The majority of females had 1–2 lifetime sexual partners (71%), whereas the highest percentage of males reported five or more sexual partners (36%) (p≤0.001). Most young people (91%) had one current sexual partner; 14% of males reported two or more (p≤0.001). Among females, 62% reported ever being pregnant and 42% ever giving birth. Males reported significantly lower rates of each, with only 19% reporting ever having a pregnant partner (p≤0.001) and 10% ever fathering a child (p≤0.001). Only 2% of females ever had a cervical cancer screening and 44% had ever been tested for a sexually transmitted infection (STI); fewer males (21%) had ever been tested for STIs (p≤0.001).

### Contraceptive awareness, attitudes, and use

Awareness of and attitudes toward contraceptive methods, by sex, are presented in Table 2. Condoms were the most widely recognized method (100%), followed by withdrawal (97% females, 100% males), the pill (96% females, 92% males), injectables (96% females, 87% males), rhythm method (92% females, 99% males), and emergency contraception (88% females, 87% males). The least known methods included diaphragms (27% females, 21% males), lactational amenorrhea method (18% females, 31% males), implants (29% females, 56% males), male sterilization (49% females, 68% males), female sterilization (65% females, 82% males), and IUDs (64% females, 48% males).

Attitudes toward modern methods were negative among many respondents, particularly males. Approximately half of males believed that women may become promiscuous with contraceptive use (34% females, 54% males, p = 0.001) and that in general birth control is risky for women (16% females, 54% males, p≤0.001), and nearly all males and many females (87% females, 99% males, p = 0.001) reported at least one modern method was unsafe for women. Nonetheless, most males and females believed children in smaller families are more likely to succeed (82% females, 96% males, p = 0.001). Overall, 63% of females and 58% of males felt at risk for unintended pregnancy.

Males were significantly more likely than females to report they had ever talked about contraception with anyone (54% females, 67% males, p = 0.03), a partner (34% females, 48% males, p = 0.02), or a friend (29% females, 43% males, p = 0.02) (data not shown). Females were more likely to say they felt supported for contraceptive use by family (31% females, 19% males, p = 0.03) or healthcare providers (55% females, 42% males, p = 0.04).

Contraceptive practices, by sex, are reported in Table 3. Almost half (46%) of females and three-quarters (74%) of males reported current contraceptive use; however, only 39% of females and 57% of males reported using a modern method. The most popular birth control method was male condoms (18% females, 48% males). For females, emergency contraceptives were the next most popular modern method (12%) followed by injectables (8%). Other methods included withdrawal (10% females, 35% males), rhythm method (12% females, 17% males), the pill (6% females, 9% males), and implants (2% females, 1% males). Overall, 27% of females and 45% of males reported condom use during last sexual intercourse (p = 0.001).

**Table 3:** Contraceptive practices, by sex.

	Total sample (N = 350)		Female (n = 250)		Male (n = 100)		P-value
	N	%	n	%	n	%	
**Current method use**							
Any modern method	154	44.0%	97	38.8%	57	57.0%	**≤0.001**
Any traditional method	36	10.3%	19	7.6%	17	17.0%	
No method	160	45.7%	134	53.6%	26	26.0%	
Female sterilization	1	0.3%	1	0.4%	0	0.0%	n/a
Male sterilization	2	0.6%	2	0.8%	0	0.0%	
Pill	23	6.6%	14	5.6%	9	9.0%	
Intrauterine device	1	0.3%	1	0.4%	0	0.0%	
Implant	7	2.0%	6	2.4%	1	1.0%	
Injectables	19	5.4%	19	7.6%	0	0.0%	
Male condom	92	26.3%	44	17.6%	48	48.0%	
Female condom	1	0.3%	1	0.4%	0	0.0%	
Diaphragm	0	0.0%	0	0.0%	0	0.0%	
Foam/jelly	2	0.6%	1	0.4%	1	1.0%	
Lactational amenorrhea method	0	0.0%	0	0.0%	0	0.0%	
Rhythm method	47	13.4%	30	12.0%	17	17.0%	
Withdrawal	61	17.4%	26	10.4%	35	35.0%	
Emergency contraception	36	10.3%	30	12.0%	6	6.0%	
Male condom use at last sexual encounter							**0.001**
*Yes*	112	32.0%	67	26.8%	45	45.0%	
*No or don’t know*	238	68.0%	183	73.2%	55	55.0%	
Would recommend current method to a friend (among contraceptive users, n = 187)^a^	146	78.1%	79	70.5%	67	89.3%	**0.002**
Reason for contraception non-use							n/a
*Respondent doesn’t want to*	73	45.6%	51	38.1%	22	84.6%	
*Side effects*	59	36.9%	54	40.3%	5	19.2%	
*Partner doesn’t want to*	50	31.3%	37	27.6%	13	50.0%	
*Doesn’t mind if gets pregnant*	38	23.8%	28	20.9%	10	38.5%	
*Currently pregnant*^b^	12	7.5%					
*Infrequent sex*	16	10.0%	14	10.4%	2	7.7%	
*Don’t feel at risk of pregnancy*	13	8.1%	12	9.0%	1	3.8%	
*Trying to get pregnant*	8	5.0%	6	4.5%	2	7.7%	
*Don’t know where to obtain*	7	4.4%	7	5.2%	0	0.0%	
*Don’t want others to know*	5	3.1%	5	3.7%	0	0.0%	
*Difficult to pay for*	3	1.9%	3	2.2%	0	0.0%	
*Difficult to get appointment*	1	0.6%	1	0.7%	0	0.0%	
*Other*	13	8.1%	12	9.0%	1	3.8%	
**Source of contraceptive method (n = 114, females contraceptive users only)**							
Pharmacy			64	56.1%			n/a
Nowhere			22	19.3%			
Private hospital/clinic			10	8.8%			
Government hospital/polyclinic			6	5.3%			
Government health center			3	2.6%			
Government health post/clinic			3	2.6%			
Other public facility			1	0.9%			
Private family planning clinic			1	0.9%			
Other private facility			1	0.9%			
Would recommend facility to a friend^a^							n/a
*Yes*			52	46.0%			
*No or don’t know*			16	14.2%			
*Didn’t go to facility*			45	39.8%			

^a^Columns may not sum to total sample due to missing data.

^b^Men not asked this question.

Bolded values indicate p<0.05; n/a—not applicable

Top reasons for non-use of contraception among females (n = 134) included side effects (40%), the respondent not wanting to (38%), the partner not wanting to (28%), and not minding if they got pregnant (21%). Top reasons for non-use of contraception among males (n = 26) included the respondent not wanting to (85%), the partner not wanting to (50%), not minding if their partner got pregnant (39%), and side effects (19%). Ten percent of females and 17% of males reported ever wanting a family planning method but were unable to get it (data not shown). Most female modern contraceptive users obtained their chosen method from a pharmacy (56%).

### Predictors of contraceptive use and unintended pregnancy

Overall, 44.0% (95% CI 38.77–49.23%, n = 154) of participants reported current modern contraceptive use (versus no method or a traditional method). In multivariable regression, males and females were significantly more likely to report modern contraception use if they had a secondary education (adjusted odds ratio (AOR) 2.1, p = 0.01), tertiary education (AOR 4.3, p = 0.01), or vocational/technical schooling (AOR 2.8, p = 0.03) (compared to primary); had ever talked with someone about contraception use (AOR 4.7, p≤0.001); felt unsupported by a healthcare provider for contraceptive use (AOR 2.2, p = 0.005); or did not feel somewhat or very likely to get pregnant accidentally (AOR 2.7, p≤0.001). There were no significant differences by sex, age group, relationship status, religion, prior birth, prior abortion, sexual debut, or other contraceptive attitude or support variables.

Overall, 45.2% (95% CI 38.99–51.41%, n = 113) of females reported their last pregnancy was unintended (versus last pregnancy intended or never pregnant). Women were more likely to experience an unintended pregnancy if they had ever given birth (AOR 6.7, p≤0.001); their sexual debut was earlier (8–14 years versus 20–24 years) (AOR 3.4, p = 0.05); or they had 3–4 lifetime sexual partners versus 1–2 (AOR 2.4, p = 0.03). Females were less likely to experience unintended pregnancy if they reported women who use contraception may become promiscuous (AOR 0.5, p = 0.03). There were no significant differences by age, education, relationship status, religion, or other contraceptive attitude or support variables, or current modern contraceptive use.

## Discussion

Unintended pregnancy was common, and despite the majority of males and females feeling at risk for future unintended pregnancy, only 44% of sexually active youth were using a modern contraceptive method. There was high awareness among young men about methods such as female sterilization (82%), male sterilization (68%), the pill (92%), injectables (87%) and condoms (100%), and close to half of male respondents were aware of methods such as IUDs (48%) and implants (56%). Despite the high levels of awareness, young men seem to be reluctant to use most methods, except condoms, due to several misconceptions about the impact of contraceptives on health and beliefs that use of contraception promotes promiscuity among women. The misconceptions around modern method safety among both young men and women in particular must be addressed. On the other hand, the high awareness and use of emergency contraceptives presents an opportunity for programmers to strengthen educational efforts around longer-acting options. The high proportion of young people aware of and using withdrawal and rhythm methods may be related to perceived dangers of modern methods. Adanu et. al conducted focus group discussions with twenty-two Ghanaian women and found that information on modern methods of contraception were available from advertisements on television and radio, friends, and clinic staff. However, many misconceptions about the side effects from using modern methods existed, which led to women turning to traditional methods [24]. Our study provides the added perspective from young men that traditional methods such as rhythm method (17%) and withdrawal (35%) are preferred. It is expedient that adolescents be empowered to use modern contraceptives, which offer more effective protection.

The 45% of females aged 18–24 in our study who experienced an unintended pregnancy is similar to the 2014 Ghana Demographic and Health Survey, which found 58% aged <20 and 33% aged 20–24 had an unintended pregnancy in the prior five years [3]. The 39% of females in our study reporting current modern contraceptive use was higher than the 2014 Demographic and Health Survey data, in which 6% of females aged <20 and 21% aged 20–24 reported modern contraceptive use [3]. We hypothesize that the difference is due to our study using a different sampling strategy than was employed in the Ghana Demographic and Health Survey, and also because our study is based only in Accra, the capital city. A study of sexual and reproductive health among women in Accra estimated that despite rapid economic development and declining fertility, the current use of modern contraception was 30% (which is closer to the estimate from our study) [24].

The finding that young men and women were significantly more likely to report modern contraceptive use if they felt unsupported by a healthcare provider was surprising. It is hard to interpret why this might be the case, since respondents were not asked about the number of interactions that led them to feel supported/unsupported. It may be that these individuals were using condoms and emergency contraception, the most popular methods reported, which do not need prescriptions in Accra or regular interactions with healthcare providers. We were also surprised that participants who believed contraception use can make women promiscuous had lower odds of unplanned pregnancy. It is possible that these respondents were particularly careful with sexual partners. Additional research is needed to understand these findings.

Contextually, these responses overall highlight the fact that contraception use is not merely an outcome of awareness, knowledge, and availability, but is influenced by complex factors including decision-making processes, negotiating abilities, and power relations. For instance, our findings indicate high contraceptive awareness among women, reluctance among men to use contraceptives due to the misconceptions about its impact on health, and beliefs about contraception promoting promiscuity among women, which are similar to previous findings [24–26]. However, use may be dependent not only on the respondent wanting to but also on their partners, as well as ease of access to services. As such, interventions must focus on building the self-efficacy of youth as well as the provision of empowerment skills.

There are several other policy recommendations to address these findings. First, efforts to increase dialogue around contraception may improve uptake among young people in Accra. A substantial proportion of participants had not talked with anyone about contraception, and having talked with someone about contraception was associated with modern contraceptive use. Further, the finding that early sexual debut was related to unintended pregnancy highlights the importance of early interventions in sexual and reproductive health education. Additionally, targeting sexual and reproductive health education interventions to people with lower levels of education and outside of the school system may help to reach young people most in need, as lower education level was associated with a decreased odds of modern contraception use. Finally, the low levels of cervical cancer screenings among females and STI testing, particularly among males, should be addressed. A study examining the impacts of adolescent school-based curriculum and peer outreach activities to increase adolescent services usage of reproductive health services in a low-income district of northern Ghana found exposure was associated with over twice the odds of using STI services among participants in intervention versus comparison communities [27].

This study has several limitations. First, these data come from a cross-sectional sample and are not representative of all young people in Accra or other urban settings in Ghana. Second, despite interviewing in private locations and with peer interviewers, participants were asked sensitive questions and some may not have answered all questions openly. Additionally, data were self-reported and there may be social desirability biases in some responses. Third, male estimates of modern contraception use were likely underestimated given that their partners may be utilizing methods unbeknownst to them.

This study provides new information about contraceptive knowledge and practices and unintended pregnancy among young people in Accra, including young men. Targeted interventions are needed to improve understanding of long-acting methods and the safety of modern methods more broadly, and consequently to increase modern contraceptive access and use.

## Supporting information

S1 Appendix Female questionnaire. (PDF)

S2 Appendix Male questionnaire. (PDF)

S1 File Data file. (DTA)

S2 File Data dictionary. (DO)
