# Supplementary material for: Contraceptive use and unintended pregnancy among young women and men in Accra, Ghana
Source: PLoS One. 2018 Aug 17;13(8):e0201663. doi: 10.1371/journal.pone.0201663 (PMC6097688; doi:10.1371/journal.pone.0201663)
Supplement: S1 Appendix — (PDF) [file pone.0201663.s001.pdf]

Participant ID: F | | |

REPRODUCTIVE HEALTH DECISION MAKING AMONG URBAN YOUTH IN GHANA:
FEMALE QUESTIONNAIRE

Interview site: ______________________________	Interviewer's ID:	
Today's Date<br>Day: [ ][ ] Month: [ ][ ] Year: [ ][ ][ ][ ]	Time Interview Started<br>Hour: [ ][ ] Minutes: [ ][ ]	Time Interview Ended<br>Hour: [ ][ ] Minutes: [ ][ ]

FINAL INTERVIEW STATUS:

Interview completed	1	Refusal	5
Partly completed	2	Consent withdrawn	6
Respondent un-contactable	3	Other (specify):	7

SCREENING QUESTIONS

Before we get started, I'd like to begin with a few questions to see if you are eligible to take part in this survey. Can you tell me:

NO.	QUESTIONS AND FILTERS	CODING CATEGORIES	SKIP
a.	Sex (observe)	Female ....1<br>Male....2	
b.	How old are you?	AGE IN YEARS: [ ][ ]	→ IF NOT 18-24, END
c.	Since this survey is about young people's access to health services for their sexual health, I need to ask you a couple of questions about sexual activity. Have you had sexual intercourse any time in the past?	YES ....1<br>NO....2	→ END
d.	When was the last time you had sexual intercourse?	TIME IN WEEKS: [ ][ ]<br>**OR**<br>TIME IN MONTHS: [ ][ ]	→ } IF >6 MO, END
e.	Are you able to understand English, Twi, or Ga?	YES ....1<br>NO....2	→ END
**REVIEW CONSENT FORM WITH PARTICIPANT**			
f.	Has the consent information been reviewed?	YES ....1<br>NO....2	
g.	Did the participant give consent to participate?	YES ....1<br>NO....2	→ END

Participant ID: | F | | | |

SECTION 1. RESPONDENT'S BACKGROUND

I'd like to ask you a bit about your background. Can you tell me:

NO.	QUESTIONS AND FILTERS	CODING CATEGORIES	SKIP
101.	Are you currently in school?	YES .... 1<br>NO 2	 → GO TO 103
102.	What level are you in?	JHS .... 1<br>SHS .... 2<br>TERTIARY .... 3<br>VOCATIONAL/TECHNICAL .... 4<br>OTHER______________ .. 96<br>(SPECIFY)	1–96 → GO TO 105
103.	If not currently going to school, have you ever attended school?	YES .... 1<br>NO 2	 → GO TO 105
104.	What is the highest level you completed?	JHS .... 1<br>SHS .... 2<br>TERTIARY .... 3<br>VOCATIONAL/TECHNICAL .... 4<br>OTHER______________ .. 96<br>(SPECIFY)	
105.	What is your religion?	CATHOLIC .... 01<br>ANGLICAN .... 02<br>METHODIST .... 03<br>PRESBYTERIAN .... 04<br>PENTACOSTAL/CHARISMATIC .... 05<br>OTHER CHRISTIAN .... 06<br>MUSLIM .... 07<br>TRADITIONAL/SPIRITUALIST .... 08<br>NO RELIGION .... 09<br>OTHER______________ .. 96<br>(SPECIFY)<br>DON'T KNOW 98	
106.	To which ethnic group do you belong?	AKAN .... 01<br>GA/DANGME .... 02<br>EWE .... 03<br>GUAN .... 04<br>MOLE-DAGBANI .... 05<br>GRUSSI .... 06<br>GRUMA .... 07<br>HAUSA .... 08<br>OTHER______________ .. 96<br>(SPECIFY)<br>DON'T KNOW 98	

Participant ID: | F | | | |

NO.	QUESTIONS AND FILTERS	CODING CATEGORIES	SKIP
107.	What is your relationship status?	MARRIED....1<br>LIVING WITH PARTNER, BUT NOT MARRIED....2<br>HAVE A STEADY PARTNER, BUT NOT LIVING TOGETHER....3<br>SEPARATED/DIVORCED....4<br>WIDOWED....5<br>SINGLE/NO STEADY PARTNER....6<br>OTHER_____...96<br>(SPECIFY)<br>DON'T KNOW....98	
108.	If you have a general health problem, where do you go for care?<br>PROBE TO IDENTIFY PLACE AND RECORD.<br><br>IF UNABLE TO DETERMINE IF HOSPITAL, HEALTH CENTER, OR CLINIC IS PUBLIC OR PRIVATE, WRITE NAME OF THE PLACE:<br>_____<br>_____<br>_____<br>_____	PUBLIC SECTOR<br>GOV'T HOSPITAL/POLYCLINIC....01<br>GOV'T HEALTH CENTER....02<br>GOV'T HEALTH POST/CLINIC....03<br>MOBILE CLINIC....04<br>OTHER PUBLIC CLINIC_____..05<br>(SPECIFY)<br>PRIVATE MEDICAL SECTOR<br>PRIVATE HOSPITAL/CLINIC....06<br>MOBILE CLINIC....07<br>PHARMACY/CHEMIST/DRUG STORE....08<br>OTHER PRIVATE MEDICAL_____09<br>(SPECIFY)<br>OTHER_____..96<br>(SPECIFY)<br>DON'T KNOW....98	
109.	What is the main reason you choose this health facility?	COST....1<br>QUALITY....2<br>PRIVACY....3<br>LOCATION....4<br>ACCEPTS HEALTH INSURANCE....5<br>OTHER_____..96<br>(SPECIFY)<br>DON'T KNOW....98	
110.	Would you go to a different health facility for a sexual health issue, such as family planning or an STI test, than you would for a general health issue?	YES....1<br>NO....2<br>DON'T KNOW....98	 → GO TO 112<br>→ GO TO 112

Participant ID: F | | |

NO.	QUESTIONS AND FILTERS	CODING CATEGORIES	SKIP
110a.	What is the main reason you would go to a different health facility for a sexual health issue?	COST ....1<br>QUALITY ....2<br>PRIVACY ....3<br>LOCATION ....4<br>ACCEPTS HEALTH INSURANCE ....5<br>OTHER________ .. 96<br>(SPECIFY)<br>DON'T KNOW 98	
111.	Where do you go for sexual health issues?<br><br>PROBE TO IDENTIFY PLACE AND RECORD.<br><br>IF UNABLE TO DETERMINE IF HOSPITAL, HEALTH CENTER, OR CLINIC IS PUBLIC OR PRIVATE, WRITE NAME OF THE PLACE:<br>________<br>________<br>________<br>________	PUBLIC SECTOR<br>GOV'T HOSPITAL/POLYCLINIC .... 01<br>GOV'T HEALTH CENTER .... 02<br>GOV'T HEALTH POST/CLINIC .... 03<br>MOBILE CLINIC .... 04<br>OTHER PUBLIC CLINIC________ .. 05<br>(SPECIFY)<br>PRIVATE MEDICAL SECTOR<br>PRIVATE HOSPITAL/CLINIC .... 06<br>MOBILE CLINIC .... 07<br>PHARMACY/CHEMIST/DRUG STORE.... 08<br>OTHER PRIVATE<br>MEDICAL________09<br>(SPECIFY)<br>OTHER________. . 96<br>(SPECIFY)<br>DON'T KNOW....98	
112.	In general, where do you think there is better quality of health care, is it public or private facilities?	PUBLIC ....1<br>PRIVATE ....2<br>NO DIFFERENCE ....3<br>DON'T KNOW 98	
112a.	Why do you think this type of facility has better quality of health care?<br><br>PROBE TO IDENTIFY EACH REASON.	SHORTER WAIT TIMES .... A<br>BETTER TRAINING/SKILLS OF STAFF .... B<br>MORE FRIENDLY STAFF .... C<br>MORE COMFORTABLE FACILITY .... D<br>CLEANER FACILITY .... E<br>MORE PRIVACY .... F<br>BETTER EQUIPTMENT/SUPPLIES .... G<br>OTHER________ .. X<br>(SPECIFY)<br>DON'T KNOW .... Z	
113.	Have you ever been tested for a sexually transmitted infection in your lifetime?<br><br>IF YES, ASK: What were you tested for?	YES________........ 1<br>(SPECIFY)<br>NO ....2<br>DON'T KNOW 98	 <br><br>→ GO TO 115<br>→ GO TO 115

NO.	QUESTIONS AND FILTERS	CODING CATEGORIES	SKIP
114.	Where did you go for this test?<br><br>PROBE TO IDENTIFY EACH PLACE AND RECORD ALL PLACES MENTIONED.<br><br>IF UNABLE TO DETERMINE IF HOSPITAL, HEALTH CENTER, OR CLINIC IS PUBLIC OR PRIVATE, WRITE NAME OF THE PLACE(S):<br>________________________<br>________________________<br>________________________<br>________________________	PUBLIC SECTOR<br>GOV'T HOSPITAL/POLYCLINIC............. A<br>GOV'T HEALTH CENTER....................... B<br>GOV'T HEALTH POST/CLINIC<br>MOBILE CLINIC .... C<br>OTHER PUBLIC<br>CLINIC______________________.... D<br>(SPECIFY)<br>PRIVATE MEDICAL SECTOR<br>PRIVATE HOSPITAL/CLINIC.................. E<br>MOBILE CLINIC .... F<br>PHARMACY/CHEMIST/DRUG STORE.... G<br>OTHER PRIVATE<br>MEDICAL_____________________.. H<br>(SPECIFY)<br>OTHER___________________________ .. X<br>(SPECIFY)<br>DON'T KNOW.. Z	
115.	Have you had a Pap smear test in your lifetime? This is a test that looks for signs that a cancer in a woman's cervix might develop.	YES ....1<br>NO..2<br>DON'T KNOW.. 98	 → GO TO 117<br>→ GO TO 117
116.	Where did you go for this test?<br><br>PROBE TO IDENTIFY EACH PLACE AND RECORD ALL PLACES MENTIONED.<br><br>IF UNABLE TO DETERMINE IF HOSPITAL, HEALTH CENTER, OR CLINIC IS PUBLIC OR PRIVATE, WRITE NAME OF THE PLACE(S):<br>________________________<br>________________________<br>________________________<br>________________________	PUBLIC SECTOR<br>GOV'T HOSPITAL/POLYCLINIC............. A<br>GOV'T HEALTH CENTER....................... B<br>GOV'T HEALTH POST/CLINIC............... C<br>MOBILE CLINIC .... D<br>OTHER PUBLIC<br>CLINIC________________________ .. E<br>(SPECIFY)<br>PRIVATE MEDICAL SECTOR<br>PRIVATE HOSPITAL/CLINIC.................. F<br>MOBILE CLINIC .... G<br>OTHER PRIVATE<br>MEDICAL_____________________ .. H<br>(SPECIFY)<br>OTHER___________________________ .. X<br>(SPECIFY)<br>DON'T KNOW.. Z	

Participant ID: F | | |

Now I would like to ask you some questions about sexual activity in order to gain a better understanding of some important life issues.

NO.	QUESTIONS AND FILTERS	CODING CATEGORIES	SKIP
117.	How old were you when you had sexual intercourse for the very first time?	AGE IN YEARS: ☐☐	
118.	In total, with how many different people have you had sexual intercourse in your lifetime?	LIFETIME NUMBER OF PARTNERS: ☐☐☐	
119.	In total, how many sexual partners do you have right now?	CURRENT NUMBER OF PARTNERS: ☐☐	
120.	The last time you had sexual intercourse, was a male condom used?	YES .... 1<br>NO .... 2<br>DON'T KNOW/DON'T REMEMBER 98	→ GO TO 122<br><br>→ GO TO 122
121.	What is the reason you didn't use a condom when you last had sexual intercourse?<br><br>PROBE TO IDENTIFY EACH REASON AND CIRCLE THE APPROPRIATE CODE(S).	RESPONDENT DIDN'T WANT TO.................. A<br>PARTNER DIDN'T WANT TO.......................... B<br>USING ANOTHER METHOD.......................... C<br>DIDN'T MIND IF GOT PREGNANT................. D<br>LESS SENSATION .... E<br>DIDN'T KNOW WHERE TO GET IT .... F<br>SHOP WOULD NOT SELL .... G<br>EMBARASSED TO BUY.................................. H<br>DIFFICULT TO PAY FOR .... I<br>DIDN'T WANT OTHERS TO KNOW .... J<br>SIDE EFFECTS .... K<br>DID NOT HAVE ONE AT THE TIME ....L<br>SEX HAPPENED SPONTANEOUSLY.............. M<br>OTHER ______________________________ .. X<br>(SPECIFY)<br>DON'T KNOW... Z	
122.	Have you ever heard of Marie Stopes clinics or Bluestar clinics?	YES, MARIE STOPES .... A<br>YES, BLUESTAR.. B<br>NO, HAVE NOT HEARD OF EITHER................ C	 <br>→ GO TO 201
123.	Have you ever been to a Marie Stopes Clinic or a Blue Star clinic?<br><br>If, yes, what for?<br>PROBE TO IDENTIFY EACH CLINIC TYPE, AND REASON(S) FOR VISIT.	YES, MARIE STOPES .... A<br>YES, BLUESTAR.. B<br>NO, HAVE NOT BEEN TO EITHER CLINIC .... C<br>DON'T KNOW... D<br><br>IF YES, REASON:<br>____<br><br>____<br>(SPECIFY)	

Participant ID: F | | |

SECTION 2: REPRODUCTIVE HISTORY

Now I would like to ask about all the pregnancies you have had during your life.

NO.	QUESTIONS AND FILTERS	CODING CATEGORIES	SKIP
201.	Have you ever given birth to a baby?	YES .... 1<br>NO 2	 → GO TO 204
202.	How many times have you given birth to a baby in your lifetime?	NUMBER OF BIRTHS: ☐☐	
203.	How old were you when your (first) child was born?	AGE IN YEARS: ☐☐	
204.	Some women lose their pregnancy spontaneously, that is they have a miscarriage. Have you ever had a miscarriage?	YES .... 1<br>NO 2	 → GO TO 206
205.	How many miscarriages have you had in your lifetime?	NUMBER OF MISCARRIAGES: ☐☐<br>DON'T KNOW 98	
206.	Women sometimes take steps to end their pregnancy, because they find themselves pregnant when they do not want to be, or when it is difficult for them to continue a pregnancy because of opposition from their husband, partner, relatives, or others. Have you ever been in a situation where you had to end your pregnancy?	YES .... 1<br>NO 2	 → GO TO 208
207.	How many pregnancies have ended this way in your lifetime?	NUMBER OF ABORTIONS: ☐☐<br>DON'T KNOW 98	
208.	Some women have stillbirths, that is, they give birth in late pregnancy to a dead child. Have you ever had a still birth?	YES .... 1<br>NO 2	 → GO TO 210
209.	How many stillbirths have you had in your lifetime?	NUMBER OF STILLBIRTHS: ☐☐<br>DON'T KNOW 98	
210.	Are you pregnant now?	YES .... 1<br>NO .... 2<br>DON'T KNOW 98	
211.	Just to make sure that I have this right: you have had in TOTAL _____ pregnancies during your life. Is that correct?	YES .... 1<br>NO 2	 → PROBE TO CORRECT 201-210
212.	**CHECK 211**	If ZERO PREGNANCIES .... 1<br>IF ONE OR MORE PREGNANCIES 2	→ GO TO 301<br>→ GO TO 213

Participant ID: | F | | | |

NO.	QUESTIONS AND FILTERS	CODING CATEGORIES	SKIP
213.	At the time you (last) became pregnant, did you want to become pregnant <u>then</u>, did you want to wait until <u>later</u>, or did you not want to have any (more) children at all?	THEN .... 1<br>LATER .... 2<br>NOT AT ALL 3	
214.	At the time you (last) became pregnant, were you using any method of family planning?	YES .... 1<br>NO 2	 → GO TO 214b
214a.	What method of family planning were you using?<br><br>PROBE TO IDENTIFY EACH TYPE AND CIRCLE ALL MENTIONED.	FEMALE STERILIZATION .... A<br>MALE STERILIZATION/VASECTOMY .... B<br>PILL .... C<br>IUD .... D<br>INJECTABLES .... E<br>IMPLANTS .... F<br>MALE CONDOM .... G<br>FEMALE CONDOM .... H<br>DIAPHRAGM .... I<br>FOAM/JELLY .... J<br>LACTATIONAL AMEN. METHOD .... K<br>RHYTHM METHOD .... L<br>WITHDRAWAL .... M<br>EMERGENCY CONTRACEPTION .... N<br>OTHER ______________ X<br>(SPECIFY)<br>DON'T KNOW .... Z	GO TO 215
214b.	Why weren't you using a method of family planning at that time?<br><br>PROBE TO IDENTIFY EACH REASON.	RESPONDENT DIDN'T WANT TO .... A<br>PARTNER DIDN'T WANT TO .... B<br>DIDN'T MIND IF GOT PREGNANT .... C<br>SIDE EFFECTS .... D<br>DIDN'T KNOW WHERE TO GET IT .... E<br>DOCTOR/NURSE WOULD NOT PRESCRIB .... F<br>DIFFICULT TO GET TRANSPORTATION .... G<br>DIFFICULT TO GET APPOINTMENT .... H<br>DIFFICULT TO PAY FOR METHOD .... I<br>DIDN'T WANT OTHERS TO KNOW .... J<br>TRYING TO GET PREGNANT .... K<br>DIDN'T FEEL AT RISK OF PREGNANCY .... L<br>INFREQUENT SEX .... M<br>OTHER REASON______________ ... X<br>(SPECIFY)<br>DON'T KNOW .... Z	
215.	Have you ever seen anyone for antenatal care (ANC) during a pregnancy?	YES .... 1<br>NO .... 2<br>DON'T KNOW 98	 → GO TO 217<br>→ GO TO 217

Participant ID: | F | | | |

NO.	QUESTIONS AND FILTERS	CODING CATEGORIES	SKIP
216.	Where did you receive antenatal care?<br><br>PROBE TO IDENTIFY EACH PLACE AND RECORD ALL PLACES MENTIONED.<br><br>IF UNABLE TO DETERMINE IF HOSPITAL, HEALTH CENTER, OR CLINIC IS PUBLIC OR PRIVATE, WRITE NAME OF THE PLACE(S):<br>________________________<br>________________________<br>________________________<br>________________________	HOME<br>RESPONDENTS HOME .... A<br>OTHER HOME .... B<br>PUBLIC SECTOR<br>GOV'T HOSPITAL/POLYCLINIC .... C<br>GOV'T HEALTH CENTER .... D<br>GOV'T HEALTH POST/CLINIC .... E<br>MOBILE CLINIC..................................... F<br>OTHER PUBLIC CLINIC ___________ .. G<br>(SPECIFY)<br>PRIVATE MEDICAL SECTOR<br>PRIVATE HOSPITAL/CLINIC .... H<br>MOBILE CLINIC .... I<br>MATERNITY HOME .... J<br>OTHER PRIVATE<br>MEDICAL ______________________K<br>(SPECIFY)<br>OTHER___________________________ .... X<br>(SPECIFY)<br>DON'T KNOW.. Z	
217.	If you have ever given birth, who assisted with the (last) delivery?<br><br>PROBE FOR THE TYPE(S) OF PERSON(S) AND RECORD ALL MENTIONED.<br><br>IF RESPONDENT SAYS NO ONE ASSISTED, PROBE TO DETERMINE WHETHER ANY ADULTS WERE PRESENT AT THE DELIVERY.	HEALTH PERSONNEL<br>DOCTOR .... A<br>NURSE/MIDWIFE .... B<br>AUXILLARY MIDWIFE .... C<br>OTHER PERSON<br>TRAINED TRADITIONAL BIRTH<br>ATTENDANT .... D<br>UNTRAINED TRAD BIRTH ATTENDANT . E<br>RELATIVE/FRIEND .... F<br>OTHER____________________________ .. X<br>(SPECIFY)<br>NO ONE.. Y<br>NEVER GAVE BIRTH...................................... G	

SECTION 3: ABORTION

Now I'd like to ask you some questions about your (last) pregnancy that ended in an abortion.

NO.	QUESTIONS AND FILTERS	CODING CATEGORIES	SKIP
301.	**CHECK 206 AND 207:**	ONE OR MORE ABORTIONS.......................... 1<br>NO ABORTIONS.. 2	→ GO TO 302<br>→ GO TO 343

Participant ID: | F | | | |

NO.	QUESTIONS AND FILTERS	CODING CATEGORIES	SKIP
302.	Why did you decide to have this abortion?	HEALTH OF RESPONDENT....A<br>RISK OF BIRTH DEFECT....B<br>NO MONEY TO TAKE CARE OF BABY ....C<br>TOO YOUNG TO HAVE CHILD....D<br>NOT READY TO BE A MOTHER....E<br>WANTED TO CONTINUE SCHOOLING ....F<br>DID NOT LOVE THE FATHER....G<br>WANTED TO DELAY CHILDBEARING ....H<br>WANTED TO CONTINUE WORKING ....I<br>DIDN'T WANT TO STAY WITH THE FATHER .J<br>WANTED TO SPACE CHILD....K<br>PARTNER DIDN'T WANT CHILD....L<br>CHILD'S SEX....M<br>BECAUSE OF RAPE ....N<br>TO AVOID SHAME....O<br>AFRAID OF PARENTS....P<br>NO ONE TO HELP ME LOOK AFTER THE CHILD ....Q<br>PARENTS INSISTED....R<br>FATHER OF CHILD DIED....S<br>PARTNER DENIED THE PREGNANCY ....T<br>CHANGE IN RELATIONSHIP WITH PARTNER U<br>OTHER______________________________ ..X<br>(SPECIFY)<br>DON'T KNOW....Z	
303.	How many months pregnant were you when you ended the pregnancy?	MONTHS PREGNANT: [ ][ ]<br>DON'T KNOW.... 98	
304.	Women sometimes take many steps to stop a pregnancy. Did you do more than one thing to end this pregnancy?	YES .... 1<br>NO.... 2	 → GO TO 318

Participant ID: | F | | | |

NO.	QUESTIONS AND FILTERS	CODING CATEGORIES	SKIP
305.	What did you first do to end this pregnancy?	DRANK MILK/COFFEE/GUINNESS/OTHER LIQUID WITH LOTS OF SUGAR ....01<br>DRANK HERBAL CONCOCTION ....02<br>DRANK OTHER HOME REMEDIES ....03<br>USED ANY HERBAL ENEMA..........................04<br>INSERTED HERB/OBJECT/OTHER SUBSTANCE IN THE VAGINA..05<br>TOOK TABLETS ....06<br>HEAVY MASSAGE ....07<br>D & C...08<br>MANUAL VACUUM ASPIRATION ....09<br>SURGICAL..10<br>INJECTION ....11<br>SALINE INSTILLATION..................................12<br>CYTOTEC TABLETS (MISOPROSTOL) ....13<br>MIFEPRISTONE TABLETS ....14<br>MIFEPRISTONE/MISOPROSTOL COMBI PK..15<br>OXYTOCIN ....16<br>CATHETER ....17<br>EXCESSIVE PHYSICAL ACTIVITY ....18<br>OTHER ______________________________ ..96<br>(SPECIFY)	06 → MARK '1' IN 306<br>13, 14, 15 → MARK '1' IN 306
306	**CHECK 305:**	TABLETS TAKEN (TABLETS (06), CYTOTEC/MISOPROSTOL (13), MIFEPRISTONE (14), OR COMBI PK (15))...................................1<br>TABLETS NOT TAKEN 2	2 → GO TO 308

307	**Can you tell me about how you took the tablets?**<br>PROBE FOR ORDER, NAME, # TAKEN OF EACH, DESCRIPTION, ROUTE.			
		Method #1	Method #2	Method #3
307a.	Name of tablets			
307b.	# of tablets taken			
307c.	Color/shape of tablets			
307d.	Route administered<br>SWALLOWED (ORALLY) . . . A<br>VAGINALLY. . . . . .B<br>RECTALLY. . . . . .C<br>IN THE CHEEK. . . . . D<br>UNDER TONGUE. . . . . .E<br>OTHER________________ . .X<br>(SPECIFY)<br>DON'T KNOW . . . . .Z			

NO.	QUESTIONS AND FILTERS	CODING CATEGORIES	SKIP
308.	Why did you decide to use this method?<br>PROBE TO IDENTIFY EACH REASON AND CIRCLE ALL REASONS MENTIONED.	COST .... A<br>HEALTH PROVIDER RECOMMENDED........ B<br>FRIEND/FAMILY RECOMMENDED .... C<br>PARTNER RECOMMENDED........ D<br>PRIVACY .... E<br>SAFETY .... F<br>LOCATION .... G<br>ONLY METHOD KNEW ABOUT........H<br>EFFECTIVENESS........I<br>OTHER______________________________ ... X<br>(SPECIFY)<br>DON'T KNOW........ Z	
309.	Did you see anyone to get this first step done?<br><br>If yes, who did you see?	HEALTH PROFESSIONAL<br>DOCTOR........01<br>NURSE/MIDWIFE........02<br>AUXILIARY MIDWIFE ....03<br>OTHER PERSON<br>PHARMACIST/CHEMIST........04<br>TRADITIONAL BIRTH ATTENDANT ....05<br>COMMUNITY HEALTH WORKER........06<br>RELATIVE/FRIEND........07<br>TRADITIONAL PRACTITIONER........08<br>OTHER ___________________________ ....96<br>(SPECIFY)<br>NO ONE........09<br>DON'T KNOW........98	 <br><br><br><br><br><br><br><br><br><br><br>→ GO TO 315<br>→ GO TO 315
310.	Where did you go to get this first step done?<br><br>IF UNABLE TO DETERMINE IF HOSPITAL, HEALTH CENTER, OR CLINIC IS PUBLIC OR PRIVATE, WRITE NAME OF THE PLACE(S):<br>__________________________<br>__________________________<br>__________________________<br>__________________________	PUBLIC SECTOR<br>GOV'T HOSPITAL/ POLYCLINIC ....01<br>GOV'T HEALTH CENTER........02<br>GOV'T HEALTH POST/CLINIC ....03<br>MOBILE CLINIC ....04<br>OTHER PUBLIC CLINIC _______________ ..05<br>(SPECIFY)<br>PRIVATE MEDICAL SECTOR<br>PRIVATE HOSPITAL/CLINIC ....06<br>MOBILE CLINIC ....07<br>MATERNITY HOME ....08<br>PHARMACY/CHEMIST/DRUG STORE........09<br>OTHER PVT.MEDICAL_________________10<br>(SPECIFY)<br>HOME<br>RESPONDENT'S HOME........11<br>OTHER HOME ....12<br>TBA'S HOME ....13<br>OTHER: _____________________________ ..96<br>(SPECIFY)<br>DON'T KNOW/DON'T REMEMBER 98	

Participant ID: | F | | | |

NO.	QUESTIONS AND FILTERS	CODING CATEGORIES	SKIP
311.	How did you hear about where you could go for this first procedure?<br><br>PROBE TO IDENTIFY ALL SOURCES AND CIRCLE ALL SOURCES MENTIONED.	PARTNER..A<br>MOTHER ....B<br>FATHER ....C<br>OTHER FAMILY MEMBER............................D<br>FRIEND ....E<br>DOCTOR OR NURSE .... F<br>PHARMACIST/CHEMIST ....G<br>ADVERTISEMENT ....H<br>OTHER ____________________________ ...X<br>(SPECIFY)<br>DON'T KNOW..Z	
312.	Why did you decide to go to this provider?<br><br>PROBE TO IDENTIFY EACH REASON AND CIRCLE ALL REASONS MENTIONED.	COST ....A<br>PRIVACY ....B<br>QUALITY..C<br>DISTANCE..D<br>ACCEPTS INSURANCE..................................E<br>WAIT TIMES .... F<br>FAMILY/FRIEND RECOMMENDED .... G<br>PARTNER RECOMMENDED.......................... H<br>ADVERTISEMENT .... I<br>OTHER ____________________________ ..X<br>(SPECIFY)<br>DON'T KNOW..Z	
313.	How much did you pay for this procedure?	GHc____________<br>(SPECIFY)<br>DON'T KNOW.....................................99998	
314.	Who paid for this procedure?<br><br>PROBE TO IDENTIFY ALL SOURCES AND CIRCLE ALL SOURCES MENTIONED.	RESPONDENT..A<br>PARTNER ....B<br>MOTHER ....C<br>FATHER ....D<br>OTHER FAMILY MEMBER............................E<br>FRIEND ....F<br>OTHER ____________________________ ...X<br>(SPECIFY)<br>NO ONE...Y	
315.	**Now I would like to talk about any problems that you may have had when you had this first thing done to stop the pregnancy.**<br>IF YES FOR EACH: Was it mild, moderate or severe?		
315a.	Did you have any bleeding?	MILD ....1<br>MODERATE ....2<br>SEVERE...3<br>DID NOT HAVE BLEEDING...........................4<br>DON'T KNOW..98	

Participant ID: | F | | | |

NO.	QUESTIONS AND FILTERS	CODING CATEGORIES	SKIP
315b.	Did you have any pain?	MILD ....1<br>MODERATE ....2<br>SEVERE ....3<br>DID NOT HAVE PAIN ....4<br>DON'T KNOW 98	
315c.	Did you get a fever?	MILD ....1<br>MODERATE ....2<br>SEVERE ....3<br>DID NOT HAVE FEVER ....4<br>DON'T KNOW 98	
315d.	Did you experience any injury or suffer a perforation?	MILD ....1<br>MODERATE ....2<br>SEVERE ....3<br>DID NOT HAVE INJURY ....4<br>DON'T KNOW 98	
315e.	Did you experience any abnormal vaginal discharge?	MILD ....1<br>MODERATE ....2<br>SEVERE ....3<br>DID NOT HAVE DISCHARGE ....4<br>DON'T KNOW 98	
316.	Did you have any other problems?<br><br>IF YES, ASK: What other problems did you have?	YES________________ .... 1<br>(SPECIFY)<br>NO ....2<br>DON'T KNOW 98	
317.	Were you given any pain relievers?	YES ....1<br>NO ....2<br>DON'T KNOW 98	
317a.	Overall, how satisfied were you with the abortion method you <u>first</u> used?	VERY SATISFIED ....1<br>SOMEWHAT SATISFIED ....2<br>SOMEWHAT DISSATISFIED ....3<br>VERY DISSATISFIED ....4<br>NOT SURE 98	
317b.	Overall, how satisfied were you with the place you <u>first</u> went for this abortion?	VERY SATISFIED ....1<br>SOMEWHAT SATISFIED ....2<br>SOMEWHAT DISSATISFIED ....3<br>VERY DISSATISFIED ....4<br>DON'T KNOW ....98<br>DIDN'T GO ANYWHERE FOR ABORTION 5	

NO.	QUESTIONS AND FILTERS	CODING CATEGORIES	SKIP
318.	What was the last thing you did to end this pregnancy?<br><br>**[OR]** What did you do to end this pregnancy?	DRANK MILK/COFFEE/GUINNESS/OTHER LIQUID WITH LOTS OF SUGAR ....01<br>DRANK HERBAL CONCOCTION ....02<br>DRANK OTHER HOME REMEDIES ....03<br>USED ANY HERBAL ENEMA..........................04<br>INSERTED HERB/OBJECT/OTHER SUBSTANCE IN THE VAGINA...05<br>TOOK TABLETS ....06<br>HEAVY MASSAGE ....07<br>D & C..08<br>MANUAL VACUUM ASPIRATION ....09<br>SURGICAL..10<br>INJECTION ....11<br>SALINE INSTILLATION..................................12<br>CYTOTEC TABLETS (MISOPROSTOL) ....13<br>MIFEPRISTONE TABLETS ....14<br>MIFEPRISTONE/MISOPROSTOL COMBI PK..15<br>OXYTOCIN ....16<br>CATHETER ....17<br>EXCESSIVE PHYSICAL ACTIVITY ....18<br>OTHER ____________________________ ..96<br>(SPECIFY)	06 → MARK '1' IN 319<br><br>13, 14, 15 → MARK '1' IN 319
319.	**CHECK 318:**	TABLETS TAKEN (TABLETS (06), CYTOTEC/MISOPROSTOL (13), MIFEPRISTONE (14), OR COMBI PK (15))..................................1<br>TABLETS NOT TAKEN 2	2 → GO TO 321

320.	**Can you tell me about how you took the tablets?**<br>PROBE FOR ORDER, NAME, # TAKEN OF EACH, DESCRIPTION, ROUTE.			
		Method #1	Method #2	Method #3
320a.	Name of tablets			
320b.	# of tablets taken			
320c.	Color/shape of tablets			
320d.	Route administered<br>SWALLOWED (ORALLY) . . . . . .A<br>VAGINALLY. . . . . B<br>RECTALLY. . . . . .C<br>IN THE CHEEK. . . . . .D<br>UNDER TONGUE. . . . . E<br>OTHER_______________ . . . . .X<br>(SPECIFY)<br>DON'T KNOW . . . . Z			

NO.	QUESTIONS AND FILTERS	CODING CATEGORIES	SKIP
321.	Why did you decide to use this method?<br><br>PROBE TO IDENTIFY EACH REASON AND CIRCLE ALL REASONS MENTIONED.	COST ....A<br>HEALTH PROVIDER RECOMMENDED........B<br>FRIEND/FAMILY RECOMMENDED ....C<br>PARTNER RECOMMENDED........D<br>PRIVACY ....E<br>SAFETY ....F<br>LOCATION ....G<br>ONLY METHOD KNEW ABOUT........H<br>EFFECTIVENESS........I<br>OTHER______________________________ ..X<br>(SPECIFY)<br>DON'T KNOW........Z	
322.	Did you see anyone to get this (last step) done?<br><br>If yes, who did you see?	HEALTH PROFESSIONAL<br>DOCTOR........01<br>NURSE/MIDWIFE........02<br>AUXILIARY MIDWIFE ....03<br>OTHER PERSON<br>PHARMACIST/CHEMIST........04<br>TRADITIONAL BIRTH ATTENDANT........05<br>COMMUNITY HEALTH WORKER........06<br>RELATIVE/FRIEND........07<br>TRADITIONAL PRACTITIONER........08<br>OTHER __________________________...96<br>(SPECIFY)<br>NO ONE........10<br>DON'T KNOW........98	 <br><br><br><br><br><br><br><br><br><br><br>→ GO TO 328<br>→ GO TO 328
323.	Where did you go to get this (last step) done?<br><br>IF UNABLE TO DETERMINE IF HOSPITAL, HEALTH CENTER, OR CLINIC IS PUBLIC OR PRIVATE, WRITE NAME OF THE PLACE(S):<br><br>__________________________<br>__________________________<br>__________________________<br>__________________________	PUBLIC SECTOR<br>GOV'T HOSPITAL/POLYCLINIC ....01<br>GOV'T HEALTH CENTER........02<br>GOV'T HEALTH POST/CLINIC ....03<br>MOBILE CLINIC ....04<br>OTHER PUBLIC CLINIC _______________..05<br>(SPECIFY)<br>PRIVATE MEDICAL SECTOR<br>PRIVATE HOSPITAL/CLINIC ....06<br>MOBILE CLINIC ....07<br>MATERNITY HOME ....08<br>PHARMACY/CHEMIST/DRUG STORE........09<br>OTHER PVT.MEDICAL_________________10<br>(SPECIFY)<br>HOME<br>RESPONDENT'S HOME........11<br>OTHER HOME ....12<br>TBA'S HOME ....13<br>OTHER______________________________ ..96<br>(SPECIFY)<br>DON'T KNOW/DON'T REMEMBER 98	

NO.	QUESTIONS AND FILTERS	CODING CATEGORIES	SKIP
324.	How did you hear about where you could go for this procedure?<br><br>PROBE TO IDENTIFY ALL SOURCES AND CIRCLE ALL MENTIONED.	PARTNER....A<br>MOTHER.... B<br>FATHER.... C<br>OTHER FAMILY MEMBER....D<br>FRIEND....E<br>DOCTOR OR NURSE.... F<br>PHARMACIST/CHEMIST....G<br>ADVERTISEMENT....H<br>OTHER ______________ . X<br>(SPECIFY)<br>DON'T KNOW....Z	
325.	Why did you decide to go to this provider?<br><br>CIRCLE ALL REASONS MENTIONED.	COST.... A<br>PRIVACY.... B<br>QUALITY.... C<br>DISTANCE.... D<br>ACCEPTS INSURANCE....E<br>WAIT TIMES.... F<br>FAMILY/FRIEND RECOMMENDED.... G<br>PARTNER RECOMMENDED.... H<br>ADVERTISEMENT.... I<br>OTHER ______________ .. X<br>(SPECIFY)<br>DON'T KNOW.... Z	
326.	How much did you pay for this procedure?	GHc____________<br>(SPECIFY)<br>DON'T KNOW.... 99998	
327.	Who paid for this procedure?<br><br>PROBE FOR ALL SOURCES AND CIRCLE ALL MENTIONED.	RESPONDENT....A<br>PARTNER....B<br>MOTHER....C<br>FATHER....D<br>OTHER FAMILY MEMBER....E<br>FRIEND....F<br>OTHER ______________ ...X<br>(SPECIFY)<br>NO ONE....Y	
328.	**Now I would like to talk about any problems that you may have had when you had this (last step) done to stop the pregnancy.** IF YES FOR EACH: Was it mild, moderate or severe?		
328a.	Did you have any bleeding?	MILD....1<br>MODERATE....2<br>SEVERE....3<br>DID NOT HAVE BLEEDING....4<br>DON'T KNOW....98	

NO.	QUESTIONS AND FILTERS	CODING CATEGORIES	SKIP
328b.	Did you have any pain?	MILD .... 1<br>MODERATE .... 2<br>SEVERE .... 3<br>DID NOT HAVE PAIN .... 4<br>DON'T KNOW 98	
328c.	Did you get a fever?	MILD .... 1<br>MODERATE .... 2<br>SEVERE .... 3<br>DID NOT HAVE FEVER .... 4<br>DON'T KNOW 98	
328d.	Did you suffer an injury or perforation?	MILD .... 1<br>MODERATE .... 2<br>SEVERE .... 3<br>DID NOT HAVE INJURY .... 4<br>DON'T KNOW 98	
328e.	Did you experience any abnormal vaginal discharge?	MILD .... 1<br>MODERATE .... 2<br>SEVERE .... 3<br>DID NOT HAVE DISCHARGE .... 4<br>DON'T KNOW 98	
329.	Did you have any other problems?<br><br>IF YES, ASK: What other problems did you have?	YES________________________ . 1<br>(SPECIFY)<br>NO .... 2<br>DON'T KNOW 98	
330.	Were you given any pain relievers?	YES .... 1<br>NO .... 2<br>DON'T KNOW 98	
331.	Did you get any treatment for any health problems you had because of the abortion?<br><br>IF YES, ASK: What kind of treatment did you receive?<br><br>PROBE FOR ALL TREATMENTS AND CIRCLE ALL TREATMENTS MENTIONED.	OPERATION .... A<br>BLOOD TRANSFUSION .... B<br>ANTIBIOTICS .... C<br>OTHER ______________________ .. X<br>(SPECIFY)<br>NO TREATMENT .... Y	 <br><br><br><br>→ GO TO 333

Participant ID: | F | | | |

NO.	QUESTIONS AND FILTERS	CODING CATEGORIES	SKIP
332.	Where did you go to get this treatment?<br><br>IF UNABLE TO DETERMINE IF HOSPITAL, HEALTH CENTER, OR CLINIC IS PUBLIC OR PRIVATE, WRITE NAME OF THE PLACE(S):<br>________________________<br>________________________<br>________________________<br>________________________	PUBLIC SECTOR<br>GOV'T HOSPITAL/ POLYCLINIC ....01<br>GOV'T HEALTH CENTER ....02<br>GOV'T HEALTH POST/CLINIC ....03<br>MOBILE CLINIC ....04<br>OTHER PUBLIC CLINIC _______________ ..05<br>(SPECIFY)<br>PRIVATE MEDICAL SECTOR<br>PRIVATE HOSPITAL/CLINIC ....06<br>MOBILE CLINIC ....07<br>MATERNITY HOME ....08<br>PHARMACY/CHEMIST/DRUG STORE ....09<br>OTHER PRIVATE<br>MEDICAL________________________ ..10<br>(SPECIFY)<br>HOME<br>RESPONDENT'S HOME.............................11<br>OTHER HOME ....12<br>TBA'S HOME ....13<br>OTHER______________________________ ..96<br>(SPECIFY)<br>DON'T KNOW..98	
333.	After 6 months, did you have any health problems as a result of this abortion?	YES ....1<br>NO...2<br>NOT YET 6 MONTHS ....3<br>DON'T KNOW..98	 → GO TO 335<br>→ GO TO 335<br>→ GO TO 335
334.	What health problems did you have?<br><br>PROBE: ANY OTHER? CIRCLE ALL MENTIONED.	ABDOMINAL PAIN.......................................A<br>STERILITY...B<br>INFECTION ....C<br>LACK OF PERIOD ....D<br>IRREGULAR PERIOD ....E<br>MORE PAINFUL PERIOD..............................F<br>STILL PREGNANT..G<br>OTHER ______________________________ . X<br>(SPECIFY)	
335.	If a doctor, chemist, or other health worker assisted with your abortion, did they talk to you before or after the abortion about family planning?	YES, BEFORE THE ABORTION .... 1<br>YES, AFTER THE ABORTION........................... 2<br>BOTH BEFORE AND AFTER THE ABORTION .. 3<br>NO... 4<br>DID NOT TALK TO HEALTH WORKER .... 5<br>DON'T KNOW...98	 <br><br>→ GO TO 337<br>→ GO TO 337<br>→ GO TO 337
336.	Did the doctor, chemist, or other health worker give you a method, prescribe a method, or refer you to a family planning clinic?	YES, GAVE METHOD.................................... 1<br>YES, PRESCRIBED A METHOD.......................2<br>YES, GAVE REFERRAL ....3<br>NO...4<br>DID NOT TALK TO HEALTH WORKER............ 5<br>DON'T KNOW.. 98	

Participant ID: | F | | | |

NO.	QUESTIONS AND FILTERS	CODING CATEGORIES	SKIP
337.	Overall, how satisfied were you with the abortion method you (last) used?	VERY SATISFIED.......... 1<br>SOMEWHAT SATISFIED.......... 2<br>SOMEWHAT DISSATISFIED.......... 3<br>VERY DISSATISFIED .... 4<br>DON'T KNOW.......... 98	
338.	Overall, how satisfied were you with the place you (last) went for this abortion?	VERY SATISFIED.......... 1<br>SOMEWHAT SATISFIED.......... 2<br>SOMEWHAT DISSATISFIED.......... 3<br>VERY DISSATISFIED .... 4<br>DON'T KNOW.......... 98<br>DIDN'T GO ANYWHERE FOR ABORTION...... 5	
338a.	Did you talk to anyone about this abortion?<br><br>PROBE TO IDENTIFY EACH PERSON RESPONDENT TALKED TO ABOUT ABORTION.	YES, PARTNER .... A<br>YES, MOTHER.......... B<br>YES, FATHER.......... C<br>YES, OTHER FAMILY .... D<br>YES, FRIEND .... E<br>YES, HEALTH CARE PROVIDER .... F<br>YES, CHEMIST.......... G<br>OTHER ______________ .X<br>(SPECIFY)<br>NO, NOBODY .... H	
339.	Do you feel that your friends supported your decision to have an abortion?	YES .... 1<br>NO.......... 2<br>DON'T KNOW/FRIENDS NOT INFORMED 98	
340.	Do you feel that your partner supported your decision to have an abortion?	YES .... 1<br>NO.......... 2<br>DON'T KNOW/PARTNER NOT INFORMED ... 98	
341.	Do you feel that your family supported your decision to have an abortion?	YES .... 1<br>NO.......... 2<br>DON'T KNOW/FAMILY NOT INFORMED 98	
342.	Do you feel that health care providers supported your decision to have an abortion?	YES .... 1<br>NO.......... 2<br>DON'T KNOW/PROVIDER NOT INFORMED.. 98	→ GO TO 351<br>→ GO TO 351<br>→ GO TO 351

IF NEVER HAD AN ABORTION:

NO.	QUESTIONS AND FILTERS	CODING CATEGORIES	SKIP
343.	Have you heard of abortion?<br><br>IF NO PROBE: That is a woman can deliberately end a pregnancy that she does not want. Have you heard about this?	YES .... 1<br>NO.......... 2	 → GO TO 401
344.	Have you heard of any place where a woman can go to get an abortion?	YES .... 1<br>NO.......... 2<br>DON'T KNOW.......... 98	 → GO TO 347<br>→ GO TO 347

Participant ID: F

NO.	QUESTIONS AND FILTERS	CODING CATEGORIES	SKIP
345.	Where is that? Any other place?<br><br>PROBE TO IDENTIFY EACH TYPE OF SOURCE AND CIRCLE THE APPROPRIATE CODE(S). MARK ALL THAT APPLY.<br><br>IF UNABLE TO DETERMINE IF HOSPITAL, HEALTH CENTER, OR CLINIC IS PUBLIC OR PRIVATE, WRITE NAME OF THE PLACE(S):<br>____________________________<br>____________________________<br>____________________________<br>____________________________	PUBLIC SECTOR<br>GOV'T HOSPITAL/POLYCLINIC................A<br>GOV'T HEALTH CENTER ....B<br>GOV'T HEALTH POST/CLINIC..................C<br>MOBILE CLINIC.......................................D<br>OTHER PUBLIC CLINIC _____________ ..E<br>(SPECIFY)<br>PRIVATE MEDICAL SECTOR<br>PRIVATE HOSPITAL/CLINIC.....................F<br>MOBILE CLINIC.......................................G<br>MATERNITY HOME ....H<br>PHARMACY/CHEMIST/DRUG STORE ......I<br>OTHER PRIVATE<br>MEDICAL_______________________...J<br>(SPECIFY)<br>HOME<br>RESPONDENT'S HOME...........................K<br>OTHER HOME...L<br>TBA'S HOME..M<br>OTHER_____________________________ ..X<br>(SPECIFY)	
346.	How did you hear about where women can go for an abortion?<br><br>CIRCLE ALL SOURCES MENTIONED.	PARTNER...A<br>MOTHER ....B<br>FATHER ....C<br>OTHER FAMILY MEMBER............................D<br>FRIEND ....E<br>DOCTOR OR NURSE ....F<br>PHARMACIST/CHEMIST ....G<br>ADVERTISEMENT ....H<br>OTHER _____________________________ ...X<br>(SPECIFY)<br>DON'T KNOW..Z	
347.	Do you think you would be supported by your friends if you decided to have an abortion?	YES ....1<br>NO...2<br>DON'T KNOW...98	
348.	Do you think you would be supported by your partner if you decided to have an abortion?	YES ....1<br>NO ....2<br>DON'T KNOW...98	
349.	Do you think you would be supported by your family if you decided to have an abortion?	YES ....1<br>NO...2<br>DON'T KNOW...98	
350.	Do you think you would be supported by a health care provider if you decided to have an abortion?	YES ....1<br>NO...2<br>DON'T KNOW...98	

Participant ID: | F | | | |

ASK TO ALL PARTICIPANTS:

NO.	QUESTIONS AND FILTERS	CODING CATEGORIES	SKIP
351.	Is abortion legal in Ghana?	YES, UNDER ALL CONDITIONS ....1<br>YES, UNDER SOME CONDITIONS ....2<br>NO, UNDER NO CONDITIONS ....3<br>DON'T KNOW..98	 <br>→ GO TO 355<br>→ GO TO 355
352.	Under what conditions is abortion legal in Ghana?<br><br>PROBE: ANYTHING ELSE? CIRCLE ALL MENTIONED.	RAPE...A<br>INCEST...B<br>LIFE OF WOMAN IN DANGER .... C<br>RISK TO PHYSICAL HEALTH OF WOMAN...... D<br>RISK TO MENTAL HEALTH OF WOMAN ....E<br>FOETAL ABNORMALITY ....F<br>DURING FIRST TRIMESTER ONLY ....G<br>THROUGH SECOND TRIMESTER.................. H<br>MOTHER MENTALLY NOT SOUND .... I<br>ALL CIRCUMSTANCES/CONDITIONS .... J<br>OTHER (SPECIFY)______________________ X<br>DON'T KNOW..Z	
353.	Can minors, or people below 18 years, legally access abortion?	YES .... 1<br>NO ....2<br>DEPENDS_________________________ .... 3<br>(SPECIFY)<br>DON'T KNOW..98	 → GO TO 355<br><br><br>→ GO TO 355
354.	Do minors, or people below 18 years, need parental consent to obtain abortion?	YES ....1<br>NO...2<br>DON'T KNOW..98	
355.	Do you think young women in general know where they can go for an abortion?	YES ....1<br>NO... 2<br>DON'T KNOW..98	

Participant ID: | F | | | |

SECTION 4: FAMILY PLANNING

Now I would like to talk about family planning - the various ways or methods that a couple can use to delay or avoid a pregnancy.

401. Which ways or methods have you heard about?

FOR METHODS NOT MENTIONED SPONTANEOUSLY, ASK:

Have you ever heard of (METHOD)?

CIRCLE CODE 1 IN 401 FOR EACH METHOD MENTIONED SPONTANEOUSLY.
THEN PROCEED DOWN COLUMN 401, READING THE NAME AND DESCRIPTION OF EACH METHOD NOT MENTIONED SPONTANEOUSLY.
CIRCLE CODE 1 IF METHOD IS SPONTANEOUSLY MENTIONED, CODE 2 IF RECOGNIZED WHEN PROMPTED, AND 3 IF NOT RECOGNIZED.
THEN, FOR EACH METHOD WITH CODE 1 OR 2 CIRCLED IN 401, ASK 402 (Have you ever used [METHOD]?).

METHOD	401: MENTION SPONTANEOUSLY **OR** RECOGNIZE	402: HAVE YOU EVER USED [METHOD]?
a. FEMALE STERILIZATION Women can have an operation to avoid having any more children.	SPONTANEOUS..........1<br>RECOGNIZE................2 → 402<br>NEITHER.....................3	HAVE YOU EVER HAD AN OPERATION TO AVOID HAVING MORE CHILDREN?<br><br>YES ....1<br>NO............................. 2
b. MALE STERILIZATION OR VASECTOMY Men can have an operation to avoid having any more children.	SPONTANEOUS..........1<br>RECOGNIZE................2 → 402<br>NEITHER.....................3	HAVE YOU EVER HAD A PARTNER WHO HAD AN OPERATION TO AVOID HAVING MORE CHILDREN?<br><br>YES ....1<br>NO............................. 2
c. PILL Women can take a pill every day to avoid becoming pregnant.	SPONTANEOUS..........1<br>RECOGNIZE................2 → 402<br>NEITHER.....................3	YES ....1<br>NO............................. 2
d. IUD Women can have a device--something called a loop or coil--placed inside their uterus by a doctor or nurse.	SPONTANEOUS..........1<br>RECOGNIZE................2 → 402<br>NEITHER.....................3	YES ....1<br>NO............................. 2
e. INJECTABLES Women can have an injection by a health provider that stops them from becoming pregnant for 1 or 3 or more months.	SPONTANEOUS..........1<br>RECOGNIZE................2 → 402<br>NEITHER.....................3	YES ....1<br>NO............................. 2

Participant ID: F | | | |

METHOD	401: MENTION SPONTANEOUSLY **OR** RECOGNIZE	402: HAVE YOU EVER USED [METHOD]?
g. MALE CONDOM Men can put a rubber sheath on their penis before sexual intercourse.	SPONTANEOUS.........1<br>RECOGNIZE...............2<br>NEITHER...................3	YES ....1<br>NO 2
h. FEMALE CONDOM Women can place a sheath in their vagina before sexual intercourse.	SPONTANEOUS.........1<br>RECOGNIZE...............2<br>NEITHER...................3	YES ....1<br>NO 2
i. DIAPHRAGM Women place a dome-shaped cup made of latex or silicone in their vagina before sexual intercourse.	SPONTANEOUS.........1<br>RECOGNIZE...............2<br>NEITHER...................3	YES ....1<br>NO 2
j. FOAM/JELLY Women insert a film, foam, gel, or suppository into their vagina that contains chemicals that stop sperm from moving.	SPONTANEOUS.........1<br>RECOGNIZE...............2<br>NEITHER...................3	YES ....1<br>NO 2
k. LACTATIONAL AMENORRHEA METHOD (LAM) Breastfeeding can reduce risk of pregnancy.	SPONTANEOUS.........1<br>RECOGNIZE...............2<br>NEITHER...................3	YES ....1<br>NO 2
l. RHYTHM METHOD Every month that a woman is sexually active she can avoid pregnancy by not having sexual intercourse on the days of the month she is most likely to get pregnant.	SPONTANEOUS.........1<br>RECOGNIZE...............2<br>NEITHER...................3	YES ....1<br>NO 2
m. WITHDRAWAL Men can be careful and pull out before climax.	SPONTANEOUS.........1<br>RECOGNIZE...............2<br>NEITHER...................3	YES ....1<br>NO 2
n. EMERGENCY CONTRACEPTION As an emergency measure after unprotected sexual intercourse, women can take special pills at any time within five days to prevent pregnancy.	SPONTANEOUS.........1<br>RECOGNIZE...............2<br>NEITHER...................3	YES ....1<br>NO 2
o. Have you heard of any other ways or methods that women or men can use to avoid pregnancy?	YES ....1<br>______________<br>(SPECIFY)<br>______________<br>(SPECIFY)<br>NO 2	 YES ....1<br>NO .... 2<br>YES .... 1<br>NO 2

NO.	QUESTIONS AND FILTERS	CODING CATEGORIES	SKIP
403.	Are you currently doing something or using any method to delay or avoid getting pregnant?	YES .... 1<br>NO 2	 → GO TO 416
404.	Which method are you using?<br><br>PROBE TO IDENTIFY EACH TYPE AND CIRCLE ALL MENTIONED.	FEMALE STERILIZATION .... A<br>MALE STERILIZATION/VASECTOMY .... B<br>PILL... C<br>IUD .... D<br>INJECTABLES .... E<br>IMPLANTS .... F<br>MALE CONDOM .... G<br>FEMALE CONDOM .... H<br>DIAPHRAGM .... I<br>FOAM/JELLY .... J<br>LACTATIONAL AMEN. METHOD................... K<br>RHYTHM METHOD .... L<br>WITHDRAWAL.. M<br>EMERGENCY CONTRACEPTION .... N<br>OTHER ______________________________ ..X<br>(SPECIFY)	
405.	The last time you obtained this method, how much did you pay in total, including the cost of the method(s) and any consultation you may have had?	GHc____________<br>(SPECIFY)<br>DON'T KNOW...................................... 99998<br>DID NOT PAY FOR METHOD................00000	

Participant ID: | F | | | |

NO.	QUESTIONS AND FILTERS	CODING CATEGORIES	SKIP
406.	Where did you get your method(s)? Any other place?<br><br>PROBE TO IDENTIFY EACH TYPE OF SOURCE AND CIRCLE THE APPROPRIATE CODE(S).<br><br>IF UNABLE TO DETERMINE IF HOSPITAL, HEALTH CENTER, OR CLINIC IS PUBLIC OR PRIVATE, WRITE NAME OF THE PLACE(S):<br>_________________________<br>_________________________<br>_________________________<br>_________________________	PUBLIC SECTOR<br>GOV'T HOSPITAL/POLYCLINIC..............A<br>GOV'T HEALTH CENTER........................B<br>GOV'T HEALTH POST/CLINIC................C<br>FAMILY PLANNING CLINIC....................D<br>MOBILE CLINIC.....................................E<br>FIELDWORKER......................................F<br>OTHER PUBLIC ________________ __ ...G<br>(SPECIFY)<br>PRIVATE MEDICAL SECTOR<br>PRIVATE HOSPITAL/CLINIC...................H<br>PRIVATE DOCTOR.................................I<br>MOBILE CLINIC.....................................J<br>PHARMACY/CHEMIST/DRUG<br>STORE ....K<br>FIELDWORKER......................................L<br>FP/PPAG CLINIC...................................M<br>MATERNITY HOME...............................N<br>OTHER PRIVATE<br>MEDICAL ______________________ ..O<br>(SPECIFY)<br>OTHER SOURCE<br>SHOP ....P<br>CHURCH..Q<br>FRIEND/RELATIVE.................................R<br>OTHER _____________________________ ..X<br>(SPECIFY)	
407.	Overall, how satisfied are you with the family planning method(s) you are using?	VERY SATISFIED...1<br>SOMEWHAT SATISFIED...............................2<br>SOMEWHAT DISSATISFIED..........................3<br>VERY DISSATISFIED ....4<br>NOT SURE 98	
408.	If you had a friend who wanted to get a family planning method, would you recommend that she use the method(s) that you are using?<br><br>IF YES OR NO: What is the reason that you would or would not recommend this method?	YES ....1<br>NO...2<br>DON'T KNOW...98<br><br>REASON: _____________________________<br><br>______________________________________<br>(SPECIFY)	
409.	Overall, how satisfied are you with the facility/facilities where you obtained your method?	VERY SATISFIED...1<br>SOMEWHAT SATISFIED...............................2<br>SOMEWHAT DISSATISFIED..........................3<br>VERY DISSATISFIED ....4<br>NOT SURE ....98<br>DID NOT GO TO FACILITY............................5	

NO.	QUESTIONS AND FILTERS	CODING CATEGORIES	SKIP
410.	If you had a friend who wanted to get a family planning method, would you recommend that she get the method at the facility where you obtained your method?<br><br>IF YES OR NO: What is the reason that you would or would not recommend this place?	YES ....1<br>NO ....2<br>DON'T KNOW ....98<br>DID NOT GO TO FACILITY ....3<br><br>REASON: ___________________________<br><br>___________________________________<br>(SPECIFY)	
411.	Did you talk to anyone about your decision to use family planning?<br><br>PROBE TO IDENTIFY EACH PERSON RESPONDENT TALKED TO ABOUT FAMILY PLANNING.	YES, PARTNER ....A<br>YES, MOTHER...B<br>YES, FATHER..C<br>YES, OTHER FAMILY ....D<br>YES, FRIEND ....E<br>YES, HEALTH CARE PROVIDER ....F<br>YES, CHEMIST...G<br>OTHER _____________________________ .X<br>(SPECIFY)<br>NO, NOBODY ....H	
412.	Do you feel that your friends support your decision to use family planning?	YES ....1<br>NO ....2<br>DON'T KNOW/FRIENDS NOT INFORMED 98	
413.	Do you feel that your partner supports your decision to use family planning?	YES ....1<br>NO ....2<br>DON'T KNOW/PARTNER NOT INFORMED ...98	
414.	Do you feel that your family supports your decision to use family planning?	YES ....1<br>NO ....2<br>DON'T KNOW/FAMILY NOT INFORMED 98	
415.	Do you feel that health care providers support your decision to use family planning?	YES ....1<br>NO ....2<br>DON'T KNOW/PROVIDER NOT INFORMED..98	→ GO TO 425<br>→ GO TO 425<br>→ GO TO 425

Participant ID: | F | | | |

NON-USERS OF FAMILY PLANNING

NO.	QUESTIONS AND FILTERS	CODING CATEGORIES	SKIP
416.	What is the reason you are not using a family planning method? Any others?<br><br>PROBE TO IDENTIFY EACH REASON AND CIRCLE THE APPROPRIATE CODE(S).	RESPONDENT DOESN'T WANT TO..............A<br>PARTNER DOESN'T WANT TO.....................B<br>DON'T MIND IF GET PREGNANT ....C<br>SIDE EFFECTS ....D<br>DON'T KNOW WHERE TO GET IT ....E<br>DOCTOR/NURSE WOULDN'T PRESCRIBE..... F<br>DIFFICULT TO GET TRANSPORTATION.........G<br>DIFFICULT TO GET APPOINTMENT ....H<br>DIFFICULT TO PAY FOR ....I<br>DON'T WANT OTHERS TO KNOW ....J<br>TRYING TO GET PREGNANT ....K<br>DON'T FEEL AT RISK OF PREGNANCY ....L<br>INFREQUENT SEX.......................................M<br>OTHER REASON_____________________....X<br>(SPECIFY)	
417.	Do you know of a place where you can obtain a method of family planning if you wanted one?	YES ....1<br>NO.. 2	→ GO TO 419
418.	Where is that? Any other place?<br><br>PROBE TO IDENTIFY EACH TYPE OF SOURCE AND CIRCLE THE APPROPRIATE CODE(S).<br><br>IF UNABLE TO DETERMINE IF HOSPITAL, HEALTH CENTER, OR CLINIC IS PUBLIC OR PRIVATE, WRITE NAME OF THE PLACE(S):<br>_________________________<br>_________________________<br>_________________________<br>_________________________	PUBLIC SECTOR<br>GOV'T HOSPITAL/POLYCLINIC..............A<br>GOV'T HEALTH CENTER.........................B<br>GOV'T HEALTH POST/CLINIC ....C<br>FAMILY PLANNING CLINIC....................D<br>MOBILE CLINIC ....E<br>FIELDWORKER......................................F<br>OTHER PUBLIC ___________________ ..G<br>(SPECIFY)<br>PRIVATE MEDICAL SECTOR<br>PRIVATE HOSPITAL/CLINIC...................H<br>PRIVATE DOCTOR.................................I<br>MOBILE CLINIC ....J<br>PHARMACY/CHEMIST/DRUG STORE ....K<br>FIELDWORKER......................................L<br>FP/PPAG CLINIC...................................M<br>MATERNITY HOME................................N<br>OTHER PVT. MEDICAL ______________O<br>(SPECIFY)<br>OTHER SOURCE<br>SHOP ....P<br>CHURCH..Q<br>FRIEND/RELATIVE.................................R<br>OTHER _____________________________ ..X<br>(SPECIFY)	

Participant ID: F | | | |

NO.	QUESTIONS AND FILTERS	CODING CATEGORIES	SKIP
419.	Do you think that you could get family planning if you wanted to use it?	YES .... 1<br>NO .... 2<br>DON'T KNOW 98	
420.	Have you ever talked to anyone about whether or not to use family planning?<br><br>PROBE TO IDENTIFY EACH PERSON RESPONDENT TALKED TO ABOUT FAMILY PLANNING.	YES, PARTNER .... A<br>YES, MOTHER .... B<br>YES, FATHER .... C<br>YES, OTHER FAMILY .... D<br>YES, FRIEND .... E<br>YES, HEALTH CARE PROVIDER .... F<br>YES, CHEMIST .... G<br>OTHER ______________ .X<br>(SPECIFY)<br>NO, NOBODY .... H	
421.	Do you think you would be supported by your friends if you decided to use family planning?	YES .... 1<br>NO .... 2<br>DON'T KNOW 98	
422.	Do you think you would be supported by your partner if you decided to use family planning?	YES .... 1<br>NO .... 2<br>DON'T KNOW 98	
423.	Do you think you would be supported by your family if you decided to use family planning?	YES .... 1<br>NO .... 2<br>DON'T KNOW 98	
424.	Do you think you would be supported by a health care provider if you decided to use family planning?	YES .... 1<br>NO .... 2<br>DON'T KNOW 98	

ASK ALL PARTICIPANTS:

NO.	QUESTIONS AND FILTERS	CODING CATEGORIES	SKIP
425.	Now I would like to ask you about a woman's risk of pregnancy.<br>From one menstrual period to the next, are there certain days when a woman is more likely to become pregnant if she has sexual intercourse?	YES .... 1<br>NO .... 2<br>DON'T KNOW 98	 → GO TO 427<br>→ GO TO 427

Participant ID: F | | | |

NO.	QUESTIONS AND FILTERS	CODING CATEGORIES	SKIP
426.	Is this time just before her period begins, during her period, right after her period has ended, or halfway between two periods?	JUST BEFORE HER PERIOD BEGINS ....1<br>DURING HER PERIOD ....2<br>RIGHT AFTER HER PERIOD HAS ENDED ....3<br>HALFWAY BETWEEN TWO PERIODS ....4<br>OTHER______________________________ 96<br>(SPECIFY)<br>DON'T KNOW 98	
427.	Do you think that a woman who is breastfeeding her baby can become pregnant?	YES ....1<br>NO ....2<br>DON'T KNOW 98	
428.	At this time in your life, how likely do you think it is that you might become pregnant accidentally or without intending to?	VERY LIKELY ....1<br>SOMEWHAT LIKELY ....2<br>NOT LIKELY ....3<br>DON'T KNOW 98	
429.	**I will now read you some statements about family planning. Please tell me if you agree or disagree with each one.**		
429a.	Family planning is women's business and a man should not have to worry about it.	AGREE ....1<br>DISAGREE ....2<br>DON'T KNOW 98	
429b.	Women who use family planning may become promiscuous.	AGREE ....1<br>DISAGREE ....2<br>DON'T KNOW 98	
429c.	Having too many children may be dangerous for a woman.	AGREE ....1<br>DISAGREE ....2<br>DON'T KNOW 98	
429d.	It is better not to have more children than we can afford.	AGREE ....1<br>DISAGREE ....2<br>DON'T KNOW 98	
429e.	Children in smaller families are more likely to succeed.	AGREE ....1<br>DISAGREE ....2<br>DON'T KNOW 98	
430.	In general, do you think that birth control is good for a woman's health or is risky for her health?	GOOD FOR HEALTH ....1<br>RISKY FOR HEALTH ....2<br>DON'T KNOW 98	

Participant ID: | F | | | |

NO.	QUESTIONS AND FILTERS	CODING CATEGORIES	SKIP
431.	In general, do you think that any of the following methods are unsafe for women's health?<br><br>READ LIST OUTLOUD AND CIRCLE THE METHODS THAT THE RESPONDENT BELIEVES ARE UNSAFE.	FEMALE STERILIZATION ....A<br>MALE STERILIZATION/VASECTOMY.............B<br>PILL...C<br>IUD ....D<br>INJECTABLES ....E<br>IMPLANTS ....F<br>MALE CONDOM ....G<br>FEMALE CONDOM ....H<br>DIAPHRAGM ....I<br>FOAM/JELLY..J<br>LACTATIONAL AMEN. METHOD..................K<br>RHYTHM METHOD.......................................L<br>WITHDRAWAL..M<br>EMERGENCY CONTRACEPTION ....N<br>OTHER ____________________________ ..X<br>(SPECIFY)	
432.	Has there ever been a time that you wanted to get a family planning method but were unable to?	YES ....1<br>NO.. 2<br>DON'T KNOW...98	 → END<br>→ END
433.	Why was it difficult?	PARTNER DIDN'T WANT TO USE IT..............A<br>DIDN'T KNOW WHERE TO GET IT ....B<br>DOCTOR/NURSE WOULD NOT PRESCRIBE ..C<br>DIFFICULT TO GET TRANSPORTATION.........D<br>DIFFICULT TO GET APPOINTMENT ....E<br>DIFFICULT TO PAY FOR ....F<br>DIDN'T WANT OTHERS TO KNOW ....G<br>OTHER REASON_______________________..X<br>(SPECIFY)<br>DON'T KNOW..Z	

END OF SURVEY
