# Supplementary material for: Contraceptive use and unintended pregnancy among young women and men in Accra, Ghana
Source: PLoS One. 2018 Aug 17;13(8):e0201663. doi: 10.1371/journal.pone.0201663 (PMC6097688; doi:10.1371/journal.pone.0201663)
Supplement: S2 Appendix — (PDF) [file pone.0201663.s002.pdf]

Participant ID: M | | |

REPRODUCTIVE HEALTH DECISION MAKING AMONG URBAN YOUTH IN GHANA: MALE QUESTIONNAIRE

Interview site: ______________________________			Interviewer's ID:			
Today's Date			Time Interview Started		Time Interview Ended	
Day:	Month:	Year:	Hour:	Minutes:	Hour:	Minutes:

FINAL INTERVIEW STATUS:

Interview completed	1	Refusal	5
Partly completed	2	Consent withdrawn	6
Respondent un-contactable	3	Other (specify):	7

SCREENING QUESTIONS

Before we get started, I'd like to begin with a few questions to see if you are eligible to take part in this survey. Can you tell me:

NO.	QUESTIONS AND FILTERS	CODING CATEGORIES	SKIP
a.	Sex (observe)	Female .... 1<br>Male 2	
b.	How old are you?	AGE IN YEARS:	→ IF NOT 18-24, END
c.	Since this survey is about young people's access to health services for their sexual health, I need to ask you a couple of questions about sexual activity. Have you had sexual intercourse any time in the past?	YES .... 1<br>NO 2	→ END
d.	When was the last time you had sexual intercourse?	TIME IN WEEKS:<br>**OR**<br>TIME IN MONTHS:	→ IF >6 MO, END
e.	Are you able to understand English, Twi, or Ga?	YES .... 1<br>NO 2	→ END
**REVIEW CONSENT FORM WITH PARTICIPANT**			
f.	Has the consent information been reviewed?	YES .... 1<br>NO 2	
g.	Did the participant give consent to participate?	YES .... 1<br>NO 2	→ END

Participant ID: | M | | | |

SECTION 1. RESPONDENT'S BACKGROUND

I'd like to ask you a bit about your background. Can you tell me:

NO.	QUESTIONS AND FILTERS	CODING CATEGORIES	SKIP
101.	Are you currently in school?	YES .... 1<br>NO 2	 → GO TO 103
102.	What level are you in?	JHS .... 1<br>SHS .... 2<br>TERTIARY .... 3<br>VOCATIONAL/TECHNICAL .... 4<br>OTHER______________ .. 96<br>(SPECIFY)	→ GO TO 105 (1, 2, 3, 4, 96)
103.	If not currently going to school, have you ever attended school?	YES .... 1<br>NO 2	 → GO TO 105
104.	What is the highest level you completed?	JHS .... 1<br>SHS .... 2<br>TERTIARY .... 3<br>VOCATIONAL/TECHNICAL .... 4<br>OTHER______________ .. 96<br>(SPECIFY)	
105.	What is your religion?	CATHOLIC .... 01<br>ANGLICAN .... 02<br>METHODIST .... 03<br>PRESBYTERIAN .... 04<br>PENTACOSTAL/CHARISMATIC .... 05<br>OTHER CHRISTIAN .... 06<br>MUSLIM .... 07<br>TRADITIONAL/SPIRITUALIST .... 08<br>NO RELIGION .... 09<br>OTHER______________ ... 96<br>(SPECIFY)<br>DON'T KNOW 98	
106.	To which ethnic group do you belong?	AKAN .... 01<br>GA/DANGME .... 02<br>EWE .... 03<br>GUAN .... 04<br>MOLE-DAGBANI .... 05<br>GRUSSI .... 06<br>GRUMA .... 07<br>HAUSA .... 08<br>OTHER______________ ... 96<br>(SPECIFY)<br>DON'T KNOW 98	

Participant ID: | M | | | |

NO.	QUESTIONS AND FILTERS	CODING CATEGORIES	SKIP
107.	What is your relationship status?	MARRIED.. 1<br>LIVING WITH PARTNER, BUT NOT MARRIED.. 2<br>HAVE A STEADY PARTNER, BUT NOT LIVING TOGETHER.. 3<br>SEPARATED/DIVORCED .... 4<br>WIDOWED .... 5<br>SINGLE/NO STEADY PARTNER .... 6<br>OTHER_____________________________...96<br>(SPECIFY)<br>DON'T KNOW... 98	
108.	If you have a general health problem, where do you go for care?<br>PROBE TO IDENTIFY PLACE AND RECORD.<br><br>IF UNABLE TO DETERMINE IF HOSPITAL, HEALTH CENTER, OR CLINIC IS PUBLIC OR PRIVATE, WRITE NAME OF THE PLACE:<br>__________________________<br>__________________________<br>__________________________<br>__________________________	PUBLIC SECTOR<br>GOV'T HOSPITAL/POLYCLINIC............. 01<br>GOV'T HEALTH CENTER....................... 02<br>GOV'T HEALTH POST/CLINIC............... 03<br>MOBILE CLINIC .... 04<br>OTHER PUBLIC CLINIC_____________ .. 05<br>(SPECIFY)<br>PRIVATE MEDICAL SECTOR<br>PRIVATE HOSPITAL/CLINIC.................. 06<br>MOBILE CLINIC .... 07<br>PHARMACY/CHEMIST/DRUG STORE.... 08<br>OTHER PRIVATE MEDICAL________________________... 09<br>(SPECIFY)<br>OTHER_____________________________. . 96<br>(SPECIFY)<br>DON'T KNOW... 98	
109.	What is the <u>main</u> reason you choose this health facility?	COST .... 1<br>QUALITY OF CARE .... 2<br>PRIVACY .... 3<br>LOCATION .... 4<br>ACCEPTS HEALTH INSURANCE .... 5<br>OTHER_____________________________ .. 96<br>(SPECIFY)<br>DON'T KNOW... 98	
110.	Would you go to a different health facility for a sexual health issue, such as family planning or an STI test, than you would for a general health issue?	YES .... 1<br>NO.. 2<br>DON'T KNOW... 98	 → GO TO 112<br>→ GO TO 112

Participant ID: M | | | |

NO.	QUESTIONS AND FILTERS	CODING CATEGORIES	SKIP
110a.	What is the main reason you would go to a different health facility for a sexual health issue?	COST .... 1<br>QUALITY OF CARE .... 2<br>PRIVACY .... 3<br>LOCATION .... 4<br>ACCEPTS HEALTH INSURANCE .... 5<br>OTHER______________ .. 96<br>(SPECIFY)<br>DON'T KNOW 98	
111.	Where do you go for sexual health issues?<br><br>PROBE TO IDENTIFY PLACE AND RECORD.<br><br>IF UNABLE TO DETERMINE IF HOSPITAL, HEALTH CENTER, OR CLINIC IS PUBLIC OR PRIVATE, WRITE NAME OF THE PLACE:<br>________________<br>________________<br>________________<br>________________	PUBLIC SECTOR<br>GOV'T HOSPITAL/POLYCLINIC .... 01<br>GOV'T HEALTH CENTER .... 02<br>GOV'T HEALTH POST/CLINIC .... 03<br>MOBILE CLINIC .... 04<br>OTHER PUBLIC CLINIC__________ .. 05<br>(SPECIFY)<br>PRIVATE MEDICAL SECTOR<br>PRIVATE HOSPITAL/CLINIC .... 06<br>MOBILE CLINIC .... 07<br>PHARMACY/CHEMIST/DRUG STORE .... 08<br>OTHER PRIVATE<br>MEDICAL______________09<br>(SPECIFY)<br>OTHER______________. . 96<br>(SPECIFY)<br>DON'T KNOW 98	
112.	In general, where do you think there is better quality of health care, is it public or private facilities?	PUBLIC .... 1<br>PRIVATE .... 2<br>NO DIFFERENCE .... 3<br>DON'T KNOW 98	
112a.	Why do you think this type of facility has better quality of health care?<br><br>PROBE TO IDENTIFY EACH REASON.	SHORTER WAIT TIMES .... A<br>BETTER TRAINING/SKILLS OF STAFF .... B<br>MORE FRIENDLY STAFF .... C<br>MORE COMFORTABLE FACILITY .... D<br>CLEANER FACILITY .... E<br>MORE PRIVACY .... F<br>BETTER EQUIPTMENT/SUPPLIES .... G<br>OTHER______________ .. X<br>(SPECIFY)<br>DON'T KNOW .... Z	
113.	Have you ever been tested for a sexually transmitted infection in your lifetime?<br><br>IF YES, ASK: What were you tested for?	YES______________........ 1<br>(SPECIFY)<br>NO .... 2<br>DON'T KNOW 98	 <br><br>→ GO TO 115<br>→ GO TO 115

NO.	QUESTIONS AND FILTERS	CODING CATEGORIES	SKIP
114.	Where did you go for this test?<br>PROBE TO IDENTIFY EACH PLACE AND RECORD ALL PLACES MENTIONED.<br><br>IF UNABLE TO DETERMINE IF HOSPITAL, HEALTH CENTER, OR CLINIC IS PUBLIC OR PRIVATE, WRITE NAME OF THE PLACE(S):<br>_________________________<br>_________________________<br>_________________________<br>_________________________	PUBLIC SECTOR<br>GOV'T HOSPITAL/POLYCLINIC............. A<br>GOV'T HEALTH CENTER........................ B<br>GOV'T HEALTH POST/CLINIC<br>MOBILE CLINIC.................................... C<br>OTHER PUBLIC<br>CLINIC__________________________. D<br>(SPECIFY)<br>PRIVATE MEDICAL SECTOR<br>PRIVATE HOSPITAL/CLINIC.................. E<br>MOBILE CLINIC.................................... F<br>PHARMACY/CHEMIST/DRUG STORE.... G<br>OTHER PRIVATE<br>MEDICAL_______________________... H<br>(SPECIFY)<br>OTHER____________________________.. Z<br>(SPECIFY)<br>DON'T KNOW.. X	

Now I would like to ask you some questions about sexual activity in order to gain a better understanding of some important life issues.

NO.	QUESTIONS AND FILTERS	CODING CATEGORIES	SKIP
115.	How old were you when you had sexual intercourse for the very first time?	AGE IN YEARS: ☐☐	
116.	In total, with how many different people have you had sexual intercourse in your lifetime?	LIFETIME NUMBER OF PARTNERS: ☐☐☐	
117.	In total, how many sexual partners do you have right now?	CURRENT NUMBER OF PARTNERS: ☐☐	
118.	The last time you had sexual intercourse, was a male condom used?	YES ....1<br>NO..2<br>DON'T KNOW/REMEMBER........................ 98	→ GO TO 120<br><br>→ GO TO 120

Participant ID: | M | | | |

NO.	QUESTIONS AND FILTERS	CODING CATEGORIES	SKIP
119.	What is the reason you didn't use a condom when you last had sexual intercourse?<br><br>PROBE TO IDENTIFY EACH REASON AND CIRCLE THE APPROPRIATE CODE(S).	RESPONDENT DIDN'T WANT TO..................A<br>PARTNER DIDN'T WANT TO..........................B<br>USING ANOTHER METHOD..........................C<br>DIDN'T MIND IF GOT PREGNANT.................D<br>LESS SENSATION ....E<br>DIDN'T KNOW WHERE TO GET IT ....F<br>SHOP WOULD NOT SELL ....G<br>EMBARASSED TO BUY..................................H<br>DIFFICULT TO PAY FOR .... I<br>DIDN'T WANT OTHERS TO KNOW ....J<br>SIDE EFFECTS ....K<br>DID NOT HAVE ONE AT THE TIME ....L<br>SEX HAPPENED SPONTANEOUSLY.............. M<br>OTHER ____________________________ .....X<br>(SPECIFY)<br>DON'T KNOW...Z	
120.	Have you ever heard of Marie Stopes clinics or Bluestar clinics?	YES, MARIE STOPES ....A<br>YES, BLUESTAR..B<br>NO, HAVE NOT HEARD OF EITHER..............C	 <br>→ GO TO 201
121.	Have you ever been to a Marie Stopes Clinic or a Blue Star clinic?<br><br>If, yes, what for?<br>PROBE TO IDENTIFY EACH CLINIC TYPE, AND REASON(S) FOR VISIT.	YES, MARIE STOPES ....A<br>YES, BLUESTAR..B<br>NO, HAVE NOT BEEN TO EITHER CLINIC......C<br>DON'T KNOW...D<br><br>IF YES, REASON:<br>____<br><br>____<br>(SPECIFY)	

SECTION 2: REPRODUCTIVE HISTORY

Now I would like to ask about any children you have had during your life. I am interested in all of the children that are biologically yours, even if they are not legally yours or do not have your last name.

NO.	QUESTIONS AND FILTERS	CODING CATEGORIES	SKIP
201.	Have you fathered any children with any woman?	YES ....1<br>NO ....2<br>DON'T KNOW.. 98	 → GO TO 204<br>→ GO TO 204
202.	How many sons and daughters have you fathered?	NUMBER OF CHILDREN: [ ][ ]<br><br>DON'T KNOW...98	
203.	How old were you when your (first) child was born?	AGE IN YEARS: [ ][ ]	

NO.	QUESTIONS AND FILTERS	CODING CATEGORIES	SKIP
204.	Women sometimes take steps to end their pregnancy, because they find themselves pregnant when they do not want to be, or when it is difficult for them to continue with their pregnancy because of opposition from their husband, partner, relatives or others. To your knowledge, has any partner of yours ever been in a situation where she did something to end a pregnancy that was biologically yours?	YES .... 1<br>NO .... 2<br>DON'T KNOW 98	 → GO TO 301<br>→ GO TO 301
205.	To your knowledge, how many pregnancies of your partners ended this way?	NUMBER OF ABORTIONS: [ ][ ]<br><br>DON'T KNOW........98	

SECTION 3: ABORTION

301.	**Check 204 AND 205**	ONE OR MORE ABORTIONS........ 1<br>NO ABORTIONS........ 2	→ GO TO 302<br>→ GO TO 311

Now I would like to ask you some questions about your partner's [last] pregnancy that ended in an abortion.

NO.	QUESTIONS AND FILTERS	CODING CATEGORIES	SKIP
302a.	What did your partner do to end this pregnancy?<br><br>CIRCLE ALL THAT APPLY.	DRANK MILK/COFFEE/GUINNESS/OTHER LIQUID WITH LOTS OF SUGAR .... A<br>DRANK HERBAL CONCOCTION .... B<br>DRANK OTHER HOME REMEDIES .... C<br>USED ANY HERBAL ENEMA........ D<br>INSERTED HERB/OBJECT/OTHER SUBSTANCE IN THE VAGINA........ E<br>TOOK TABLETS........ F<br>HEAVY MASSAGE........ G<br>D & C........ H<br>MANUAL VACUUM ASPIRATION .... I<br>SURGICAL........ J<br>INJECTION........ K<br>SALINE INSTILLATION........ L<br>CYTOTEC TABLETS (MISOPROSTOL) .... M<br>MIFEPRISTONE TABLETS........ N<br>MIFEPRISTONE/MISOPROSTOL COMBI PK O<br>OXYTOCIN........ P<br>CATHETER........ Q<br>EXCESSIVE PHYSICAL ACTIVITY .... R<br>OTHER ______________________ X<br>(SPECIFY)<br>DON'T KNOW........ Z	

NO.	QUESTIONS AND FILTERS	CODING CATEGORIES	SKIP
302b.	Did your partner see anyone to get this done?<br><br>IF YES, ASK: Who did she see?	HEALTH PROFESSIONAL<br>DOCTOR .... A<br>NURSE/MIDWIFE .... B<br>AUXILIARY MIDWIFE .... C<br>OTHER PERSON<br>PHARMACIST/CHEMIST .... D<br>TRADITIONAL BIRTH ATTENDANT .... E<br>COMMUNITY HEALTH WORKER .... F<br>RELATIVE/FRIEND .... G<br>TRADITIONAL PRACTITIONER .... H<br>OTHER __________________________ X<br>(SPECIFY)<br>NO ONE............ J<br>DON'T KNOW............ Z	 <br><br><br><br><br><br><br><br><br><br><br>→ GO TO 306<br>→ GO TO 306
303.	Where did she go to get this done?<br><br>PROBE TO IDENTIFY EACH PLACE AND CIRCLE THE APPROPRIATE CODE(S).<br><br>IF UNABLE TO DETERMINE IF HOSPITAL, HEALTH CENTER, OR CLINIC IS PUBLIC OR PRIVATE, WRITE NAME OF THE PLACE(S):<br>__________________________<br>__________________________<br>__________________________<br>__________________________	PUBLIC SECTOR<br>GOV'T HOSPITAL/POLYCLINIC .... A<br>GOV'T HEALTH CENTER .... B<br>GOV'T HEALTH POST/CLINIC............ C<br>MOBILE CLINIC............ D<br>OTHER PUBLIC CLINIC<br>________________________________... E<br>(SPECIFY)<br>PRIVATE MEDICAL SECTOR<br>PRIVATE HOSPITAL/CLINIC .... F<br>MOBILE CLINIC............ G<br>MATERNITY HOME .... H<br>PHARMACY/CHEMIST/DRUG STORE .... I<br>OTHER PRIVATE<br>MEDICAL________________________ . J<br>(SPECIFY)<br>HOME<br>RESPONDENT'S HOME............ K<br>OTHER HOME .... L<br>TBA'S HOME............ M<br>OTHER____________________________ _ . X<br>(SPECIFY)<br>DON'T KNOW............ Z	

NO.	QUESTIONS AND FILTERS	CODING CATEGORIES	SKIP
304.	How did you and your partner hear about where she could go for this procedure?<br><br>PROBE TO IDENTIFY ALL SOURCES AND CIRCLE ALL SOURCES MENTIONED.	PARTNER .... A<br>MOTHER .... B<br>FATHER .... C<br>OTHER FAMILY MEMBER .... D<br>FRIEND .... E<br>DOCTOR OR NURSE .... F<br>PHARMACIST/CHEMIST .... G<br>ADVERTISEMENT .... H<br>OTHER ____________ ... X<br>(SPECIFY)<br>DON'T KNOW .... Z	
305.	At the time your partner got pregnant, were you using any method of family planning?	YES .... 1<br>NO 2	 → GO TO 306a
306.	What method of family planning were you or your partner using?<br><br>PROBE TO IDENTIFY EACH TYPE AND CIRCLE ALL MENTIONED.	FEMALE STERILIZATION .... A<br>MALE STERILIZATION/VASECTOMY .... B<br>PILL .... C<br>IUD .... D<br>INJECTABLES .... E<br>IMPLANTS .... F<br>MALE CONDOM .... G<br>FEMALE CONDOM .... H<br>DIAPHRAGM .... I<br>FOAM/JELLY .... J<br>LACTATIONAL AMEN. METHOD .... K<br>RHYTHM METHOD .... L<br>WITHDRAWAL .... M<br>EMERGENCY CONTRACEPTION .... N<br>OTHER ____________ X<br>(SPECIFY)<br>DON'T KNOW .... Z	GO TO 307

NO.	QUESTIONS AND FILTERS	CODING CATEGORIES	SKIP
306a.	Why weren't you using a method of family planning at that time?<br><br>PROBE TO IDENTIFY EACH REASON.	RESPONDENT DIDN'T WANT TO..................A<br>PARTNER DIDN'T WANT TO..........................B<br>DIDN'T MIND IF PARTNER GOT PREGNANT .C<br>SIDE EFFECTS ....D<br>DIDN'T KNOW WHERE TO GET IT ....E<br>DOCTOR OR NURSE WOULD NOT PRESCRIBE OR SELL....................................F<br>DIFFICULT TO GET TRANSPORTATION FOR SELF OR PARTNER ....G<br>DIFFICULT TO GET APPT FOR SELF OR PARTNER...H<br>DIFFICULT TO PAY FOR METHOD.................I<br>DON'T WANT OTHERS TO KNOW ....J<br>TRYING TO GET PREGNANT ....K<br>DIDN'T FEEL AT RISK OF PREGNANCY..........L<br>INFREQUENT SEX ....M<br>OTHER REASON______________________...X<br>(SPECIFY)<br>DON'T KNOW...Z	
307	Overall, how satisfied were you with the abortion method your partner used?	VERY SATISFIED... 1<br>SOMEWHAT SATISFIED............................... 2<br>SOMEWHAT DISSATISFIED.......................... 3<br>VERY DISSATISFIED .... 4<br>NOT SURE 98	
307a.	Overall, how satisfied were you with the place your partner went for this abortion?	VERY SATISFIED... 1<br>SOMEWHAT SATISFIED............................... 2<br>SOMEWHAT DISSATISFIED.......................... 3<br>VERY DISSATISFIED .... 4<br>DON'T KNOW..98<br>DIDN'T GO ANYWHERE FOR ABORTION...... 5	
308.	Did you support your partner's decision to have an abortion?<br><br>IF YES OR NO: What is the reason you did or did not support this decision?	YES ....1<br>NO..2<br>DON'T KNOW.. 98<br><br>REASON: _____________________________<br><br>___________________________________<br>(SPECIFY)	

NO.	QUESTIONS AND FILTERS	CODING CATEGORIES	SKIP
308a.	Did you talk to anyone about this abortion?<br><br>PROBE TO IDENTIFY EACH PERSON RESPONDENT TALKED TO ABOUT ABORTION.	YES, PARTNER ....A<br>YES, MOTHER...B<br>YES, FATHER...C<br>YES, OTHER FAMILY ....D<br>YES, FRIEND ....E<br>YES, HEALTH CARE PROVIDER ....F<br>YES, CHEMIST...G<br>OTHER ______________________________ .X<br>(SPECIFY)<br>NO, NOBODY ....H	
309.	Do you feel that your family supported this decision to have an abortion?	YES .... 1<br>NO...2<br>DON'T KNOW/FAMILY NOT INFORMED 98	
309a.	Do you feel that your friends supported this decision to have an abortion?	YES .... 1<br>NO...2<br>DON'T KNOW/FRIENDS NOT INFORMED .. 98	
310.	Do you feel that health care providers supported this decision to have an abortion?	YES ....1<br>NO...2<br>DON'T KNOW/ PROVIDER NOT<br>INFORMED. 98	→ GO TO 319<br>→ GO TO 319<br><br>→ GO TO 319

IF NEVER HAD AN ABORTION

NO.	QUESTIONS AND FILTERS	CODING CATEGORIES	SKIP
311.	Have you heard of abortion?<br><br>IF NO PROBE: That is, a woman can deliberately end a pregnancy that she does not want. Have you heard about this?	YES ....1<br>NO...2	 → GO TO 401
312.	Have you heard of any place where a woman can go to get an abortion?	YES ....1<br>NO...2<br>DON'T KNOW.. 98	 → GO TO 315<br>→ GO TO 315

NO.	QUESTIONS AND FILTERS	CODING CATEGORIES	SKIP
313.	Where is that? Any other place?<br><br>PROBE TO IDENTIFY EACH TYPE OF SOURCE AND CIRCLE THE APPROPRIATE CODE(S). MARK ALL THAT APPLY.<br><br>IF UNABLE TO DETERMINE IF HOSPITAL, HEALTH CENTER, OR CLINIC IS PUBLIC OR PRIVATE, WRITE NAME OF THE PLACE(S):<br>____________________________<br>____________________________<br>____________________________<br>____________________________	PUBLIC SECTOR<br>GOV'T HOSPITAL/POLYCLINIC................A<br>GOV'T HEALTH CENTER ....B<br>GOV'T HEALTH POST/CLINIC.................. C<br>MOBILE CLINIC....................................... D<br>OTHER PUBLIC CLINIC _____________ ..E<br>(SPECIFY)<br>PRIVATE MEDICAL SECTOR<br>PRIVATE HOSPITAL/CLINIC.....................F<br>MOBILE CLINIC.......................................G<br>MATERNITY HOME ....H<br>PHARMACY/CHEMIST/DRUG STORE ......I<br>OTHER PRIVATE<br>MEDICAL________________________....J<br>(SPECIFY)<br>HOME<br>RESPONDENT'S HOME............................K<br>OTHER HOME...L<br>TBA'S HOME..M<br>OTHER_____________________________ ..X<br>(SPECIFY)	
314.	How did you hear about where women can go for an abortion?<br><br>CIRCLE ALL SOURCES MENTIONED	PARTNER...A<br>MOTHER ....B<br>FATHER ....C<br>OTHER FAMILY MEMBER............................D<br>FRIEND..E<br>DOCTOR OR NURSE .... F<br>PHARMACIST/CHEMIST ....G<br>ADVERTISEMENT ....H<br>OTHER _____________________________ .X<br>(SPECIFY)<br>DON'T KNOW..Z	
315.	Do you think you would be supported by your friends if you decided to have an abortion?	YES ....1<br>NO...2<br>DON'T KNOW... 98	
316.	Do you think you would support your partner if she decided to have an abortion?	YES ....1<br>NO...2<br>DON'T KNOW... 98	
317.	Do you think you would be supported by your family if you decided to have an abortion?	YES .... 1<br>NO...2<br>DON'T KNOW... 98	
318.	Do you think you would be supported by a health care provider if you decided to have an abortion?	YES ....1<br>NO...2<br>DON'T KNOW... 98	

Participant ID: | M | | | |

ASK ALL PARTICIPANTS:

NO.	QUESTIONS AND FILTERS	CODING CATEGORIES	SKIP
319.	Is abortion legal in Ghana?	YES, UNDER ALL CONDITIONS ....1<br>YES, UNDER SOME CONDITIONS ....2<br>NO, UNDER NO CONDITIONS ....3<br>DON'T KNOW... 98	 <br>→ GO TO 323<br>→ GO TO 323
320.	Under what conditions is abortion legal in Ghana?<br><br>PROBE: ANYTHING ELSE? CIRCLE ALL MENTIONED.	RAPE...A<br>INCEST..B<br>LIFE OF WOMAN IN DANGER ....C<br>RISK TO PHYSICAL HEALTH OF WOMAN......D<br>RISK TO MENTAL HEALTH OF WOMAN ....E<br>FOETAL ABNORMALITY...............................F<br>DURING FIRST TRIMESTER ONLY ....G<br>THROUGH SECOND TRIMESTER..................H<br>MOTHER MENTALLY NOT SOUND ....I<br>ALL CIRCUMSTANCES/CONDITIONS ....J<br>OTHER_____________________________ ..X<br>DON'T KNOW...Z	
321.	Can minors, or people below 18 years, legally access abortion?	YES ....1<br>NO...2<br>DEPENDS__________________________ .... 3<br>(SPECIFY)<br>DON'T KNOW... 98	 → GO TO 323<br><br><br>→ GO TO 323
322.	Do minors, or people below 18 years, need parental consent to obtain abortion?	YES ....1<br>NO...2<br>DON'T KNOW... 98	
323.	Do you think young women in general know where they can go for an abortion?	YES ....1<br>NO...2<br>DON'T KNOW... 98	

Participant ID: M

SECTION 4: FAMILY PLANNING

Now I would like to talk about family planning - the various ways or methods that a couple can use to delay or avoid a pregnancy.

401. Which ways or methods have you heard about?

FOR METHODS NOT MENTIONED SPONTANEOUSLY, ASK:

Have you ever heard of (METHOD)?

CIRCLE CODE 1 IN 401 FOR EACH METHOD MENTIONED SPONTANEOUSLY.
THEN PROCEED DOWN COLUMN 401, READING THE NAME AND DESCRIPTION OF EACH METHOD NOT MENTIONED SPONTANEOUSLY.
CIRCLE CODE 1 IF METHOD IS SPONTANEOUSLY MENTIONED, CODE 2 IF RECOGNIZED WHEN PROMPTED, AND 3 IF NOT RECOGNIZED.
THEN, FOR METHOD B, G, I, AND M, ASK 402 (Have you ever used [METHOD]?) IF 401 HAS CODE 1 OR 2 CIRCLED.

METHOD	401: MENTION SPONTANEOUSLY **OR** RECOGNIZE	402: HAVE YOU EVER USED [METHOD]?
a. FEMALE STERILIZATION Women can have an operation to avoid having any more children.	SPONTANEOUS..........1<br>RECOGNIZE................2<br>NEITHER....................3	
b. MALE STERILIZATION OR VASECTOMY Men can have an operation to avoid having any more children.	SPONTANEOUS..........1<br>RECOGNIZE................2<br>NEITHER....................3<br>(1, 2 → 402)	HAVE YOU EVER HAD AN OPERATION TO AVOID HAVING MORE CHILDREN?<br><br>YES ....1<br>NO............................ 2
c. PILL Women can take a pill every day to avoid becoming pregnant.	SPONTANEOUS..........1<br>RECOGNIZE................2<br>NEITHER....................3	
d. IUD Women can have a device--something called a loop or coil--placed inside their uterus by a doctor or nurse.	SPONTANEOUS..........1<br>RECOGNIZE................2<br>NEITHER....................3	

Participant ID: M

METHOD	401: MENTION SPONTANEOUSLY **OR** RECOGNIZE	402: HAVE YOU EVER USED [METHOD]?
e. INJECTABLES Women can have an injection by a health provider that stops them from becoming pregnant for 1 or 3 or more months.	SPONTANEOUS..........1<br>RECOGNIZE................2<br>NEITHER.....................3	
f. IMPLANTS Women can have several small rods placed in their upper arm by a doctor or nurse which can prevent pregnancy for one or more years.	SPONTANEOUS..........1<br>RECOGNIZE................2<br>NEITHER.....................3	
g. MALE CONDOM Men can put a rubber sheath on their penis before sexual intercourse.	SPONTANEOUS..........1 ⎫<br>RECOGNIZE................2 ⎭ →<br>NEITHER.....................3	YES ....1<br>NO............................. 2
h. FEMALE CONDOM Women can place a sheath in their vagina before sexual intercourse.	SPONTANEOUS..........1<br>RECOGNIZE................2<br>NEITHER.....................3	
i DIAPHRAGM Women place a dome-shaped cup made of latex or silicone in their vagina before sexual intercourse.	SPONTANEOUS..........1<br>RECOGNIZE................2<br>NEITHER.....................3	
j. FOAM/JELLY Women insert a film, foam, gel, or suppository into their vagina that contains chemicals that stop sperm from moving.	SPONTANEOUS..........1<br>RECOGNIZE................2<br>NEITHER.....................3	
k. LACTATIONAL AMENORRHEA METHOD (LAM) Breastfeeding can reduce risk of pregnancy.	SPONTANEOUS..........1<br>RECOGNIZE................2<br>NEITHER.....................3	
l. RHYTHM METHOD Every month that a woman is sexually active she can avoid pregnancy by not having sexual intercourse on the days of the month she is most likely to get pregnant.	SPONTANEOUS..........1 ⎫<br>RECOGNIZE................2 ⎭ →<br>NEITHER.....................3	YES ....1<br>NO............................. 2
m. WITHDRAWAL Men can be careful and pull out before climax.	SPONTANEOUS..........1 ⎫<br>RECOGNIZE................2 ⎭ →<br>NEITHER.....................3	YES ....1<br>NO............................. 2
n. EMERGENCY CONTRACEPTION As an emergency measure after unprotected sexual intercourse, women can take special pills at any time within five days to prevent pregnancy.	SPONTANEOUS..........1<br>RECOGNIZE................2<br>NEITHER.....................3	
o. Have you heard of any other ways or methods that women or men can use to avoid pregnancy?	YES............................1 →<br>__________<br>(SPECIFY)<br>__________<br>(SPECIFY)<br>NO 2	YES ....1<br>NO............................. 2<br><br>YES .... 1<br>NO............................. 2

Participant ID: | M | | | |

NO.	QUESTIONS AND FILTERS	CODING CATEGORIES	SKIP
403.	Are you and your partner doing something or using any method to avoid or prevent a pregnancy?	YES ....1<br>NO ....2<br>DON'T KNOW 98	 → GO TO 411<br>→ GO TO 411
404.	What method are you or your partner using?<br><br>PROBE TO IDENTIFY EACH TYPE AND CIRCLE ALL MENTIONED.	FEMALE STERILIZATION ....A<br>MALE STERILIZATION/VASECTOMY ....B<br>PILL....C<br>IUD ....D<br>INJECTABLES ....E<br>IMPLANTS ....F<br>MALE CONDOM ....G<br>FEMALE CONDOM ....H<br>DIAPHRAGM ....I<br>FOAM/JELLY....J<br>LACTATIONAL AMEN. METHOD....K<br>RHYTHM METHOD ....L<br>WITHDRAWAL.... M<br>EMERGENCY CONTRACEPTION .... N<br>OTHER ______________ X<br>(SPECIFY)	
405.	Overall, how satisfied are you with the family planning method(s) you are using?	VERY SATISFIED....1<br>SOMEWHAT SATISFIED....2<br>SOMEWHAT DISSATISFIED....3<br>VERY DISSATISFIED .... 4<br>NOT SURE 98	
406.	If you had a friend who wanted to get a family planning method, would you recommend that he use the method(s) that you are using?<br><br>IF YES OR NO: What is the reason that you <u>would</u> or <u>would not</u> recommend this method?	YES .... 1<br>NO.... 2<br>DON'T KNOW.... 98<br><br>REASON: ______________<br><br>______________<br>(SPECIFY)	
407.	Did you talk to anyone about your decision to use family planning?<br><br>PROBE TO IDENTIFY EACH PERSON RESPONDENT TALKED TO ABOUT FAMILY PLANNING.	YES, PARTNER ....A<br>YES, MOTHER....B<br>YES, FATHER....C<br>YES, OTHER FAMILY ....D<br>YES, FRIEND ....E<br>YES, HEALTH CARE PROVIDER ....F<br>YES, CHEMIST....G<br>OTHER ______________ .X<br>(SPECIFY)<br>NO, NOBODY ....H	
408.	Do you feel that your friends support your decision to use family planning?	YES ....1<br>NO....2<br>DON'T KNOW/FRIENDS NOT INFORMED .. 98	

Participant ID: | M | | | |

NO.	QUESTIONS AND FILTERS	CODING CATEGORIES	SKIP
409.	Do you feel that your family supports your decision to use family planning?	YES .... 1<br>NO .... 2<br>DON'T KNOW/FAMILY NOT INFORMED 98	
410.	Do you feel that health care providers support your decision to use family planning?	YES .... 1<br>NO .... 2<br>DON'T KNOW/PROVIDER NOT INFORMED.98	→ GO TO 416<br>→ GO TO 416<br>→ GO TO 416

NON-USERS OF FAMILY PLANNING

NO.	QUESTIONS AND FILTERS	CODING CATEGORIES	SKIP
411.	What is the reason you are not using a family planning method? Any others?<br><br>PROBE TO IDENTIFY EACH REASON AND CIRCLE THE APPROPRIATE CODE(S).	RESPONDENT DOESN'T WANT TO .... A<br>PARTNER DOESN'T WANT TO .... B<br>DON'T MIND IF PARTNER GETS PREGNANT C<br>SIDE EFFECTS .... D<br>DON'T KNOW WHERE TO GET IT .... E<br>DOCTOR/NURSE WOULD NOT PRESCRIBE .. F<br>DIFFICULT TO GET TRANSPORTATION .... G<br>DIFFICULT TO GET APPOINTMENT .... H<br>DIFFICULT TO PAY FOR .... I<br>DON'T WANT OTHERS TO KNOW .... J<br>TRYING TO GET PREGNANT .... K<br>DON'T FEEL AT RISK OF PREGNANCY .... L<br>INFREQUENT SEX........ M<br>OTHER REASON______________________..X<br>(SPECIFY)<br>DON'T KNOW........ Z	
411a.	Have you ever talked to anyone about whether or not to use family planning?<br><br>PROBE TO IDENTIFY EACH PERSON RESPONDENT TALKED TO ABOUT FAMILY PLANNING.	YES, PARTNER .... A<br>YES, MOTHER........ B<br>YES, FATHER........ C<br>YES, OTHER FAMILY .... D<br>YES, FRIEND .... E<br>YES, HEALTH CARE PROVIDER .... F<br>YES, CHEMIST........ G<br>OTHER ______________________________ .X<br>(SPECIFY)<br>NO, NOBODY .... H	
412.	Do you think you would be supported by your friends if you decided to use family planning?	YES .... 1<br>NO........ 2<br>DON'T KNOW........ 98	
413.	Do you think you would be supported by your partner if you decided to use family planning?	YES .... 1<br>NO........ 2<br>DON'T KNOW........ 98	
414.	Do you think you would be supported by your family if you decided to use family planning?	YES .... 1<br>NO........ 2<br>DON'T KNOW........ 98	

Participant ID: | M | | | |

NO.	QUESTIONS AND FILTERS	CODING CATEGORIES	SKIP
415.	Do you think you would be supported by a health care provider if you decided to use family planning?	YES .... 1<br>NO .... 2<br>DON'T KNOW 98	

ASK ALL PARTICIPANTS:

NO.	QUESTIONS AND FILTERS	CODING CATEGORIES	SKIP
416.	Now I would like to ask you about a woman's risk of pregnancy.<br>From one menstrual period to the next, are there certain days when a woman is more likely to become pregnant if she has sexual intercourse?	YES .... 1<br>NO .... 2<br>DON'T KNOW 98	 → GO TO 418<br>→ GO TO 418
417.	Is this time just before her period begins, during her period, right after her period has ended, or halfway between two periods?	JUST BEFORE HER PERIOD BEGINS .... 1<br>DURING HER PERIOD .... 2<br>RIGHT AFTER HER PERIOD HAS ENDED .... 3<br>HALFWAY BETWEEN TWO PERIODS .... 4<br>OTHER______________ 96<br>(SPECIFY)<br>DON'T KNOW 98	
418.	Do you think that a woman who is breastfeeding her baby can become pregnant?	YES .... 1<br>NO .... 2<br>DON'T KNOW 98	
419.	**I will now read you some statements about family planning. Please tell me if you agree or disagree with each one.**		
419a.	Family planning is women's business and a man should not have to worry about it.	AGREE .... 1<br>DISAGREE .... 2<br>DON'T KNOW 98	
419b.	Women who use family planning may become promiscuous.	AGREE .... 1<br>DISAGREE .... 2<br>DON'T KNOW 98	
419c.	Having too many children may be dangerous for a woman.	AGREE .... 1<br>DISAGREE .... 2<br>DON'T KNOW 98	
419d.	It is better not to have more children than we can afford.	AGREE .... 1<br>DISAGREE .... 2<br>DON'T KNOW 98	
419e.	Children in smaller families are more likely to succeed.	AGREE .... 1<br>DISAGREE .... 2<br>DON'T KNOW 98	

NO.	QUESTIONS AND FILTERS	CODING CATEGORIES	SKIP
420.	At this time in your life, how likely do you think it is that your partner might become pregnant accidentally or without intending to?	VERY LIKELY .... 1<br>SOMEWHAT LIKELY .... 2<br>NOT LIKELY .... 3<br>DON'T KNOW 98	
421.	In general, do you think that family planning is good for a woman's health or is risky for her health?	GOOD FOR HEALTH .... 1<br>RISKY FOR HEALTH .... 2<br>DON'T KNOW 98	
422.	In general, do you think that any of the following family planning methods are unsafe for women's health?<br><br>READ LIST OUTLOUD AND CIRCLE THE METHODS THAT THE RESPONDENT BELIEVES ARE UNSAFE.	FEMALE STERILIZATION .... A<br>MALE STERILIZATION/VASECTOMY .... B<br>PILL .... C<br>IUD .... D<br>INJECTABLES .... E<br>IMPLANTS .... F<br>MALE CONDOM .... G<br>FEMALE CONDOM .... H<br>DIAPHRAGM .... I<br>FOAM/JELLY .... J<br>LACTATIONAL AMEN. METHOD .... K<br>RHYTHM METHOD .... L<br>WITHDRAWAL .... M<br>EMERGENCY CONTRACEPTION .... N<br>OTHER ______________ ..X<br>(SPECIFY)	
423.	Do you know of a place where a person can get condoms?	YES .... 1<br>NO 2	→ GO TO 425

NO.	QUESTIONS AND FILTERS	CODING CATEGORIES	SKIP
424.	Where is that? Any other place?<br>PROBE TO IDENTIFY EACH TYPE OF SOURCE AND CIRCLE THE APPROPRIATE CODE.<br><br>IF UNABLE TO DETERMINE IF HOSPITAL, HEALTH CENTER, OR CLINIC IS PUBLIC OR PRIVATE, WRITE NAME OF THE PLACE(S):<br><br>_________________________<br>_________________________<br>_________________________<br>_________________________	PUBLIC SECTOR<br>GOV'T HOSPITAL/POLYCLINIC .... A<br>GOV'T HEALTH CENTER .... B<br>GOV'T HEALTH POST/CHPS .... C<br>FAMILY PLANNING CLINIC..................... D<br>MOBILE CLINIC .... E<br>FIELDWORKER .... F<br>OTHER PUBLIC ___________________ .. G<br>(SPECIFY)<br>PRIVATE MEDICAL SECTOR<br>PRIVATE HOSPITAL/CLINIC .... H<br>PRIVATE DOCTOR .... I<br>PHARMACY/CHEMIST/DRUG STORE ..... J<br>FP/PPAG CLINIC..................................... K<br>MATERNITY HOME .... L<br>OTHER PVT. MEDICAL ______________ N<br>(SPECIFY)<br>OTHER SOURCE<br>SHOP .... O<br>CHURCH.. P<br>COMMUNITY VOLUNTEER .... Q<br>FRIEND/RELATIVE .... R<br>DRINKING SPOT..................................... S<br>OTHER _____________________________ ... X<br>(SPECIFY)	
425.	If you wanted to, could you yourself get a condom?	YES .... 1<br>NO .... 2<br>DON'T KNOW... 98	
426.	Has there ever been a time that you wanted to get a family planning method but were unable to?	YES .... 1<br>NO .... 2<br>DON'T KNOW... 98	 → END<br>→ END
427.	Why was it difficult?	PARTNER DIDN'T WANT TO USE IT.............. A<br>DIDN'T KNOW WHERE TO GET IT .... B<br>DOCTOR OR NURSE WOULD NOT<br>PRESCRIBE OR SELL .... C<br>DIFFICULT TO GET TRANSPORTATION......... D<br>DIFFICULT TO GET APPOINTMENT .... E<br>DIFFICULT TO PAY FOR .... F<br>DIDN'T WANT OTHERS TO KNOW .... G<br>OTHER REASON______________________ .. X<br>(SPECIFY)<br>DON'T KNOW... Z	

END OF SURVEY
